# Cetylpyridinium chloride triggers paraptosis to suppress pancreatic tumor growth via the ERN1-MAP3K5-p38 pathway

**DOI:** 10.1016/j.isci.2024.110598

**Published:** 2024-07-26

**Authors:** Hu Tang, Fangquan Chen, Wanli Gao, Xiutao Cai, Zhi Lin, Rui Kang, Daolin Tang, Jiao Liu

**Affiliations:** 1 DAMP Laboratory, The Third Affiliated Hospital, Guangzhou Medical University, Guangzhou, Guangdong 510150, China; 2 Department of Pediatrics, The Third Xiangya Hospital, Central South University, Changsha, Hunan 410013, China; 3 Department of Surgery, UT Southwestern Medical Center, Dallas, TX 75390, USA

**Keywords:** Molecular biology, Cell biology, Cancer

## Abstract

Pancreatic ductal adenocarcinoma (PDAC) is a highly aggressive solid malignancy with low 5-year survival and limited treatment options. We conducted an unbiased screening using FDA-approved drug and demonstrated that cetylpyridinium chloride (CPC), a component commonly found in mouthwash and known for its robust bactericidal and antifungal attributes, exhibits anticancer activity against human PDAC cells. CPC inhibited PDAC cell growth and proliferation by inducing paraptosis, rather than apoptosis. Mechanistically, CPC induced paraptosis through the initiation of endoplasmic reticulum stress, leading to the accumulation of misfolded proteins. Subsequently, the endoplasmic reticulum stress to nucleus signaling 1 (ERN1)-mitogen-activated protein kinase kinase kinase 5 (MAP3K5)-p38 mitogen-activated protein kinase (MAPK) signaling pathway was activated, ultimately culminating in the induction of paraptosis. *In vivo* experiments, including those involving patient-derived xenografts, orthotopic models, and genetically engineered mouse models of PDAC, provided further evidence of CPC’s effectiveness in suppressing the growth of pancreatic tumors.

## Highlights

• CPC induces paraptosis in PDAC cells
• ER stress is required for CPC-induced paraptosis
• The p38 MAPK pathway plays a crucial role in CPC-induced paraptosis
• CPC demonstrates effective inhibition of PDAC tumors *in vivo*

## Graphical abstract

**Figure:**
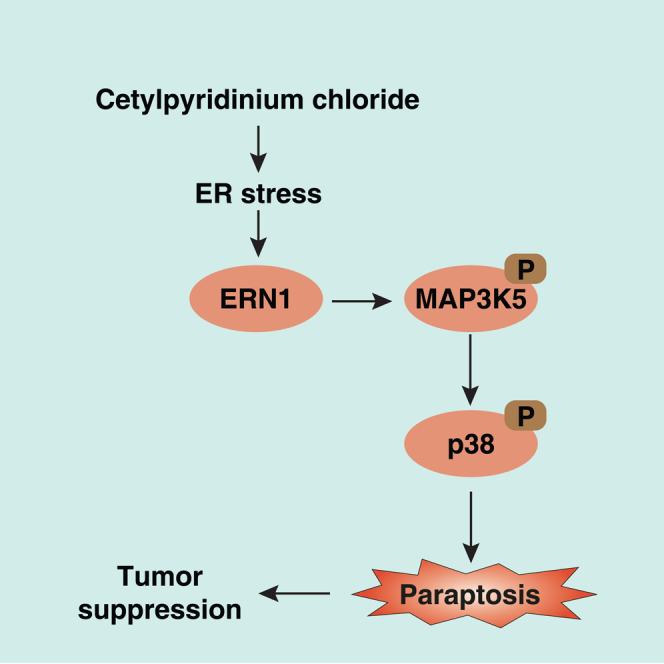

## Introduction

Pancreatic ductal adenocarcinoma (PDAC) represents one of the most fatal forms of cancer, projected to surpass colorectal cancer and become the second-leading cause of cancer-related mortality by 2040, following lung cancer.^1^ Despite progress in the 5-year overall survival rate for PDAC patients, increasing from 3% in the 1970s to 13% in 2024, these improvements remain significantly lower compared to those for other types of tumors.^2^ The primary factor contributing to this predicament is the advanced stage at which PDAC is typically diagnosed, mainly due to the absence of specific tumor markers, the lack of evident symptoms in early stages, and the challenges associated with tumor imaging.^3^ PDAC exhibits an extremely aggressive nature, often resulting in distant metastases alongside concurrent local perineural and vascular infiltration.^4^^,^^5^ As a result, the majority of PDAC patients are not eligible for radical surgical interventions, with only a limited proportion, typically ranging from 10% to 20%, meeting the criteria for such procedures.^6^ For nonsurgical patients, systemic therapy, often combined with chemotherapy, forms the cornerstone of treatment. However, the challenging pancreatic tumor microenvironment has hindered the efficacy of chemotherapy, targeted therapy, and immunotherapy.^7^^,^^8^ Therefore, there is a pressing need for novel antitumor agents to address current therapeutic limitations in PDAC.

Cetylpyridinium chloride (CPC) is a quaternary ammonium compound and ionic detergent often used as a preservative in oral care products.^9^ It is widely employed in over-the-counter items, such as mouthwash and toothpaste. CPC exerts its bactericidal effect by interacting with bacteria, replacing calcium and magnesium ions on the surface of the bacterial outer membrane with positively charged pyridine ions.^10^ This process leads to changes in intracellular osmotic pressure and the activation of ribonuclease, ultimately resulting in cellular autolysis. At higher concentrations, CPC can directly damage the cell membrane, causing the leakage of cytoplasmic contents and the impairment of proteins and nucleic acids.^11^ This damage extends to lysing the cell wall. Recent research has unveiled additional functions of CPC in inhibiting tumor growth.^12^^,^^13^^,^^14^ For example, CPC, either alone or in combination with temozolomide, can trigger apoptosis in certain solid cancers, including lung and colon cancer and glioblastoma.^12^^,^^14^^,^^15^ Yet its capacity to induce non-apoptotic cell death remains uncertain.

Paraptosis, a form of regulated cell death, involves cytoplasmic vacuole formation due to endoplasmic reticulum (ER) and mitochondria dilation.^16^ This process relies on *de novo* protein synthesis, susceptible to inhibition by cycloheximide (CHX). ER stress from misfolded protein accumulation is pivotal in paraptosis initiation. Natural compounds, such as elaiophylin, curcumin, and celastrol are recognized paraptosis inducers, showing promise in tumor therapy research.^17^^,^^18^^,^^19^ Consequently, paraptosis induction emerges as a potential strategy in antitumor therapy.^20^

In this study, we screened clinical used drugs on human PDAC cells and identified CPC as a potent anticancer agent for inducing paraptosis. We uncovered the role of the ER stress to nucleus signaling 1 (ERN1) mitogen-activated protein kinase kinase kinase 5 (MAP3K5)-p38 mitogen-activated protein kinase (MAPK) pathway in mediating CPC-induced paraptosis. Additionally, we demonstrated the efficacy of CPC in three PDAC mouse models.

## Results

### CPC suppresses PDAC cell growth

**Figure 1:**
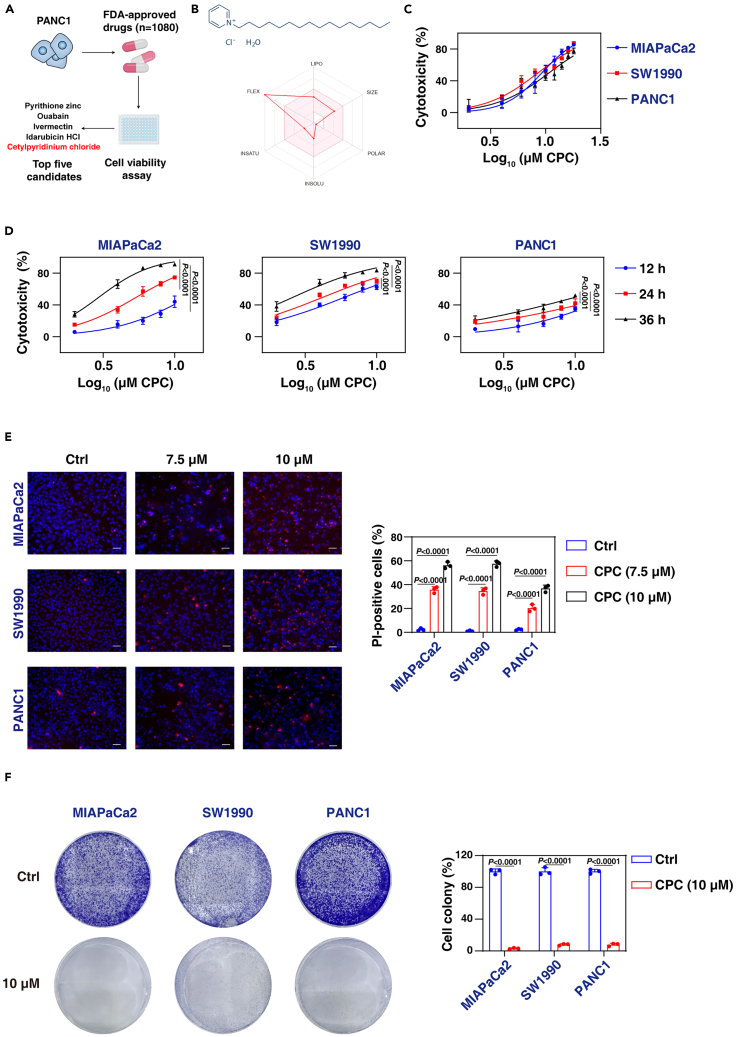
Cytotoxic effects of cetylpyridinium chloride on pancreatic cancer cells (A) Flowchart of drug screening strategy: PANC1 cells were exposed to 1,080 different FDA-approved drugs (each at a concentration of 10 μM) sourced from Selleck Chemicals in a 96-well plate for 24 h. Cell viability was assessed using a CCK8 kit. Cetylpyridinium chloride (CPC) emerged as one of the top 5 candidates with the highest inhibitory activity on cell growth, and it had not been previously studied in pancreatic cancer. (B) Chemical formula of CPC and its ADME parameters. (C) Cytotoxicity of MIAPaCa2, SW1990, and PANC1 cells following treatment with CPC for 24 h. (D) Cytotoxicity of MIAPaCa2, SW1990, and PANC1 cells following treatment with CPC for 12, 24, and 36 h. (E) Representative Hoechst 33342 and propidium iodide (PI) staining images of PDAC cells treated with CPC (7.5 μM and 10 μM) for 24 h. Scale bar: 200 μm. Quantification of PI-positive cells is shown. (F) MIAPaCa2, SW1990, and PANC1 cells were treated with CPC (7.5 μM) for 24 h, and colony formation assays were conducted as described in the Materials and Methods. Surviving colonies were counted, and representative images were captured at 2 weeks. Data are presented as the mean ± SD from three independent experiments. *p* values were calculated using two-way ANOVA (D), one-way ANOVA (E), or unpaired two-tailed Student’s t tests (F).

To identify potential agents for suppressing PDAC cell growth, we performed a screening using a drug library consisting of 1,080 Food and Drug Administration (FDA)-approved drugs. The PANC1 cell line was selected as the screening model due to its harboring the *KRAS-G12D* mutation, which is the most common gene mutation driving human PDAC.^21^ Our screening identified pyrithione zinc, ouabain, ivermectin, idarubicin HCl, and CPC as the top five candidates for inhibiting PDAC growth (Figure 1A). Among these candidates, there is currently no research on the effects of CPC on PDAC. Therefore, we focused our subsequent experiments on CPC.

“Absorption, distribution, metabolism, and excretion” (ADME) is an abbreviation in pharmacokinetics and pharmacology that describes the disposition of a pharmaceutical compound within an organism. To assess the ADME properties of CPC, we utilized an online assay website (http://www.swissadme.ch/index.php) and obtained supportive evidence suggesting that CPC demonstrates favorable drug pharmacokinetics (Figure 1B). In addition to PANC1 cells, CPC also demonstrated dose- or time-dependent cytotoxicity in MIAPaCa2 (*KRAS-G12C*) and SW1990 (*KRAS-G12D*) cells (Figures 1C and 1D). Subsequently, propidium iodide (PI) staining confirmed that CPC inhibited cell viability and effectively disrupted the integrity of plasma membranes in PANC1, MIAPaCa2, and SW1990 cells (Figure 1E). Furthermore, following CPC treatment, the surviving PDAC cells exhibited a loss of long-term cell viability, as evidenced by a clone formation assay (Figure 1F).

Collectively, these findings highlight CPC as a previously unrecognized anticancer agent in PDAC cells.

### CPC induces non-apoptotic cell death

**Figure 2:**
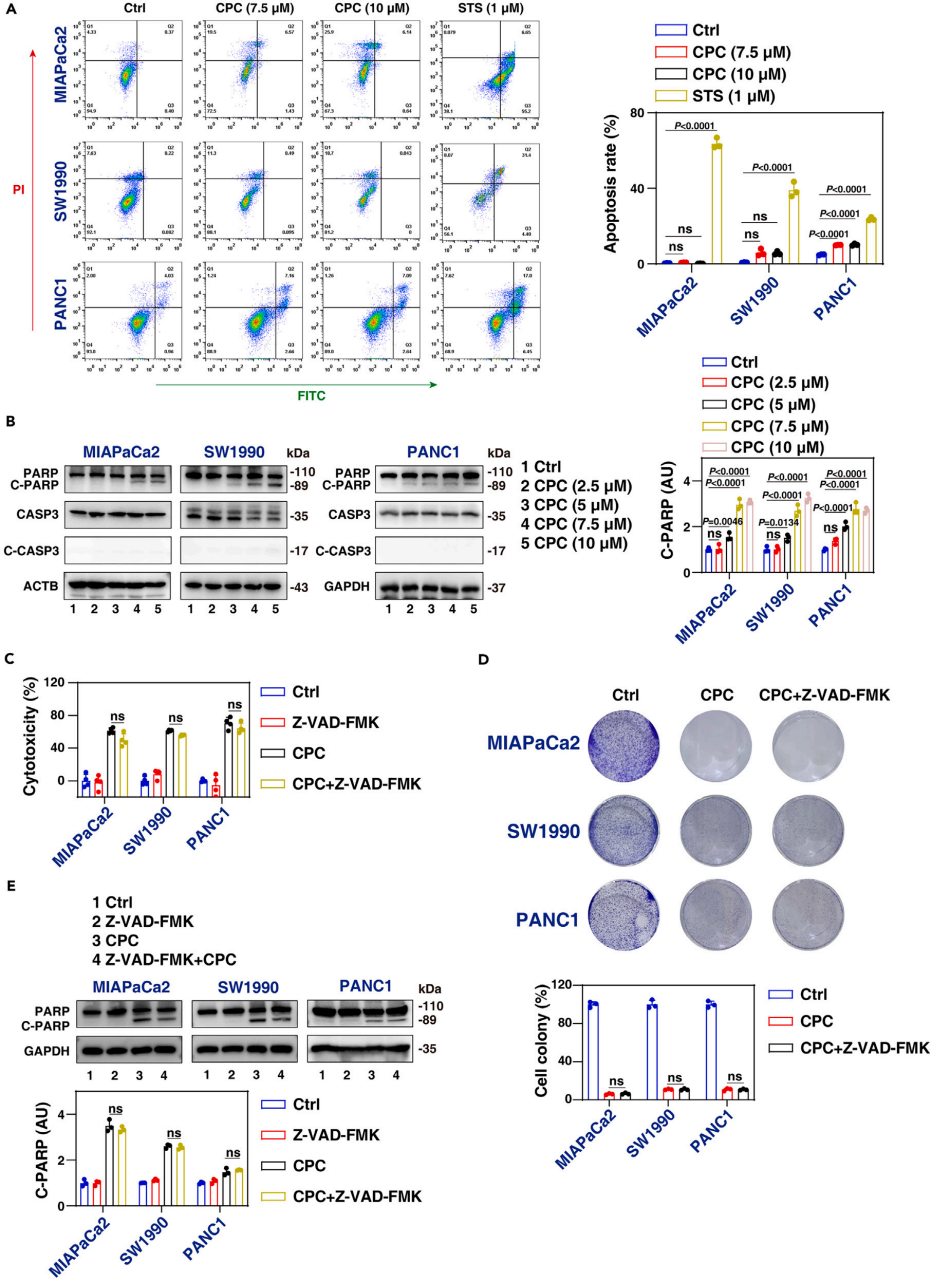
CPC induces non-apoptotic cell death in pancreatic cancer cells (A) Flow cytometry analysis of apoptosis after treating cells with CPC (7.5 and 10 μM) or staurosporine (STS; 1 μM) for 24 h. (B) Western blot analysis of the indicated protein expression in MIAPaCa2, SW1990, and PANC1 cells after treatment with CPC for 24 h. (C) Cytotoxicity of MIAPaCa2, SW1990, and PANC1 cells after treatment with CPC (7.5 μM) in the absence or presence of Z-VAD-FMK (20 μM) for 24 h. (D) MIAPaCa2, SW1990, and PANC1 cells were treated with CPC (7.5 μM) in the absence or presence of Z-VAD-FMK (20 μM) for 24 h, and colony formation assays were conducted as described in the Materials and Methods. Surviving colonies were counted, and representative images were captured at 2 weeks. (E) Western blot of protein expression in MIAPaCa2, SW1990, and PANC1 cells after treatment with CPC (7.5 μM) in the absence or presence of Z-VAD-FMK (20 μM) for 24 h. Data are presented as mean ± SD. Statistical significance was analyzed using one-way ANOVA with Dunnett’s multiple comparisons test (A–E). See also Figures S1.

Previous studies have shown that CPC exerts its anticancer effects by inhibiting mitochondrial complex I, resulting in the inhibition of mitochondrial respiration and adenosine triphosphate (ATP) production.^22^ This disruption of mitochondrial function can induce apoptosis. Our primary aim was to explore whether CPC could trigger apoptosis in PDAC cells, building on these prior findings. To achieve this, we employed a flow cytometry assay utilizing annexin V and PI double staining. This assay revealed that CPC only triggered a small number of annexin V-positive cells in MIAPaCa2, SW1990, and PANC1 cells (Figure 2A), indicating a limited induction of apoptosis. In contrast, a positive control, the classical apoptosis activator staurosporine, increased the number of annexin V-positive cells (Figure 2A).

To further evaluate apoptosis activation, we conducted western blot analyses to examine the expression of apoptosis-related effectors or substrates, specifically caspase 3 (CASP3) and poly (ADP-ribose) polymerase 1 (PARP1), following CPC treatment. After 24 h of CPC treatment at concentrations ranging from 5 to 7.5 μM, only PARP1 underwent cleavage, while the apoptosis effector CASP3 remained unaltered (Figure 2B), suggesting that CPC may induce PARP1 cleavage through a non-apoptotic mechanism. In contrast, the positive control staurosporine induced the generation of both cleaved PARP1 and cleaved CASP3 (Figure S1A).

Additionally, we investigated the impact of the pan-caspase inhibitor Z-VAD-FMK on CPC-induced growth inhibition. Z-VAD-FMK reversed staurosporine-induced cytotoxicity, yet it did not alter the growth inhibition or cytotoxicity induced by CPC (Figures 2C, 2D, and S1B). This suggests that the mechanism of cytotoxicity induced by CPC might be different from that of a classical apoptosis inducer. We also observed that the cleavage of PARP1 caused by CPC was not effectively blocked by Z-VAD-FMK (Figure 2E). These findings further confirm that cleaved PARP1 can occur through a caspase-independent mechanism, as previously reported.^23^

### CPC induces the formation of cytoplasmic vacuoles

**Figure 3:**
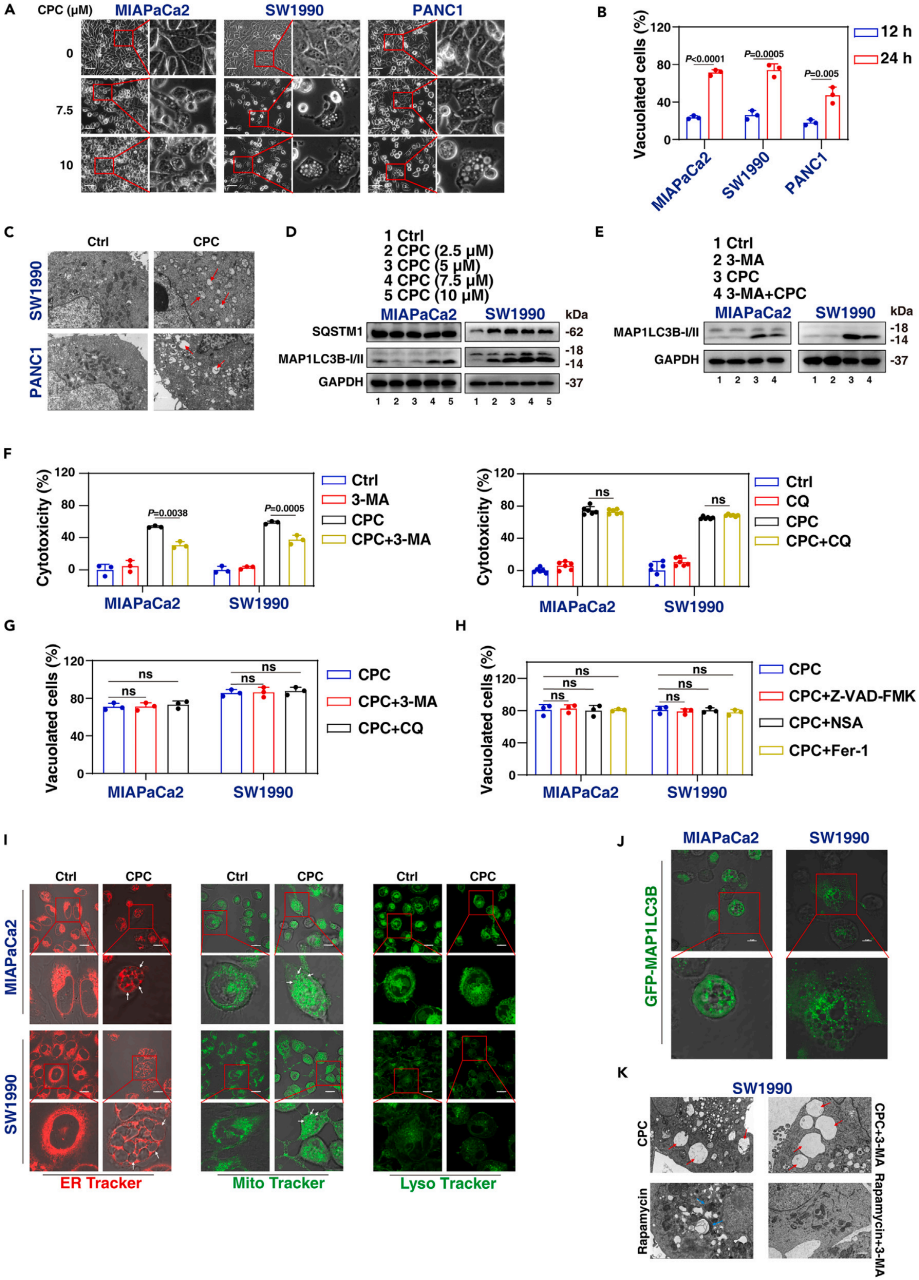
The cell vacuoles induced by CPC originated from the endoplasmic reticulum and mitochondria (A) Phase-contrast microscope analysis of cell morphology in MIAPaCa2, SW1990, and PANC1 cells after treatment with CPC (7.5 and 10 μM) for 24 h. Scale bar: 50 μm. (B) Light microscopy analysis of cell morphology in MIAPaCa2, SW1990, and PANC1 cells after treatment with CPC (7.5 μM) for 12 or 24 h. Scale bar: 50 μm. (C) Transmission electron microscopy analysis of cell ultrastructure in SW1990 and PANC1 cells following treatment with CPC (7.5 μM) for 24 h. Scale bar: 0.5 μm. (D) Western blot analysis of the expression of indicated proteins in MIAPaCa2 and SW1990 cells after treatment with CPC for 24 h. (E) Western blot analysis of the expression of indicated proteins in MIAPaCa2 and SW1990 cells after treatment with CPC (7.5 μM) in the absence or presence of 3-methyladenine (3-MA; 3 mM) for 24 h. (F) Cytotoxicity of MIAPaCa2 and SW1990 cells after treatment with CPC (7.5 μM) in the absence or presence of 3-MA (3 mM) or chloroquine (CQ; 20 μM) for 24 h. (G) Light microscopy analysis of cell morphology in MIAPaCa2 and SW1990 cells after treatment with CPC (7.5 μM) in the absence or presence of 3-MA (3 mM) or CQ (20 μM) for 24 h, followed by quantitative assessment. (H) Light microscopy analysis of cell morphology in MIAPaCa2 and SW1990 cells after treatment with CPC (7.5 μM) in the absence or presence of Z-VAD-FMK (20 μM), necrosulfonamide (NSA; 2 μM) or ferrostatin-1 (Fer-1; 1 μM) for 24 h, followed by quantitative assessment. (I) MIAPaCa2 and SW1990 cells were exposed to CPC (7.5 μM) for 24 h. ER membrane, mitochondrial, and lysosomal structures were stained and analyzed using ER-Tracker Red, MitoTracker Green, and LysoTracker Green, respectively. Scale bar: 10 μm. (J) MIAPaCa2 and SW1990 cells were exposed to CPC (7.5 μM) for 24 h, and staining with GFP-MAP1LC3B lentivirus. Scale bar: 10 μm. (K) Transmission electron microscopy analysis of cell ultrastructure was conducted in SW1990 cells following treatment with CPC (7.5 μM) or rapamycin (5 μM) in the absence or presence of 3-MA (3 mM) for 24 h. Red arrows indicate single-membrane vacuoles, while blue arrows indicate double-membrane vacuoles. Scale bar: 0.5 μm. Data are presented as mean ± SD. The proportion of cells displaying vacuoles was scored by visually examining a minimum of 100 cells and the data are presented as mean ± SD from three independent experiments (A, B, G, and H). *p* values were calculated using one-way ANOVA (F, G, and H) or unpaired two-tailed Student’s t tests (B). See also Figures S2.

Under phase-contrast microscopy examination, we observed a distinct phenomenon in CPC-treated pancreatic cancer cells, characterized by the formation and accumulation of extensive cytoplasmic vacuoles (Figure 3A). The cytoplasmic vacuolization observed in CPC-treated cells was not present in cells undergoing cell death induced by staurosporine, hydrogen peroxide (H_2_O_2_), or RSL3 (Figure S2A). The size and number of cytoplasmic vacuoles were found to be dependent on both the duration and dosage of CPC treatment (Figures 3A, 3B, and S2B). To gain deeper insights into this process, we utilized transmission electron microscopy to investigate ultrastructural features, which unveiled the presence of single-membrane vacuoles surrounding intact nuclei in PDAC cells treated with CPC (Figure 3C).

Given that autophagy, a lysosomal degradation process, frequently results in cell vacuolation,^24^^,^^25^^,^^26^^,^^27^ we investigated the potential association between CPC-induced vacuoles and heightened autophagy. We analyzed the expression of the autophagosome marker microtubule-associated protein 1 light chain 3 beta (MAP1LC3B)^28^ and observed a dose-dependent conversion of MAP1LC3B-I to MAP1LC3B-II in response to CPC treatment (Figure 3D). However, the degradation of sequestosome 1 (SQSTM1), a receptor and substrate of autophagic degradation,^29^^,^^30^^,^^31^ was not observed in response to CPC treatment (Figure 3D). Furthermore, key proteins involved in autophagy, such as autophagy-related 5 (ATG5) and autophagy-related 7 (ATG7),^32^ were not upregulated by CPC treatment (Figure S2C).

To further investigate the role of autophagy in CPC-induced cell death, we employed 3-methyladenine (3-MA) and chloroquine (CQ) as early and late-phase autophagy inhibitors, respectively.^26^^,^^33^ The western blot results showed that 3-MA reversed the accumulation of MAP1LC3B-II induced by rapamycin (Figure S2D). Additionally, CQ enhanced the accumulation of both MAP1LC3B-II and SQSTM1, confirming the efficacy of late-phase autophagy inhibitors (Figure S2E). However, 3-MA partially rescued CPC-induced cell death, whereas CQ did not (Figure 3F). Furthermore, 3-MA partially prevented the accumulation of MAP1LC3B-II induced by CPC (Figure 3E). The addition of either CQ or 3-MA did not block the formation of CPC-induced vacuoles (Figures 3G and S2F). The knockdown of *ATG7* also failed to affect CPC-induced cytotoxicity and vacuole formation (Figures S2G–S2I). Additionally, pretreatment with Z-VAD-FMK (a pan-caspase inhibitor), ferrostatin-1 (a ferroptosis inhibitor^34^), or necrosulfonamide (NSA; a necroptosis inhibitor) failed to inhibit CPC-induced cytotoxicity and vacuolation in PDAC cells (Figures 3H, S2J, and S2K). As a positive control, ferrostatin-1 and necrosulfonamide effectively restored the compromised cytotoxicity caused by RSL3 and H_2_O_2_, respectively (Figure S2L). Moreover, in comparison to positive controls, CPC did not alter the expression of critical proteins, such as glutathione peroxidase 4 (GPX4) degradation and phosphorylation of mixed lineage kinase domain like pseudokinase (MLKL), associated with ferroptosis and necroptosis (Figure S2M). Thus, CPC induces a distinct form of cell death.

To investigate the origin of vacuoles observed in CPC-treated cells, we performed staining experiments using specific fluorescent probes: MitoTracker for mitochondria, LysoTracker for lysosomes, and ER-Tracker for the ER membrane. We observed dilation of the ER membrane and swelling of the mitochondria, both of which co-localized with the vacuole, while the lysosome showed no change (Figure 3I). In contrast, staurosporine treatment did not induce similar ER or mitochondrial alterations (Figure S2N). Furthermore, the analysis of GFP-MAP1LC3B co-localization with vacuoles demonstrated no overlap (Figure 3J), and transmission electron microscopy observations revealed that 3-MA did not hinder the formation of CPC-induced single-membrane vacuoles but effectively inhibited rapamycin-induced autophagic vesicle formation (Figure 3K).

Collectively, these findings indicate that the vacuoles induced by CPC may originate from the ER and mitochondria.

### CPC induces ER stress-related paraptosis

**Figure 4:**
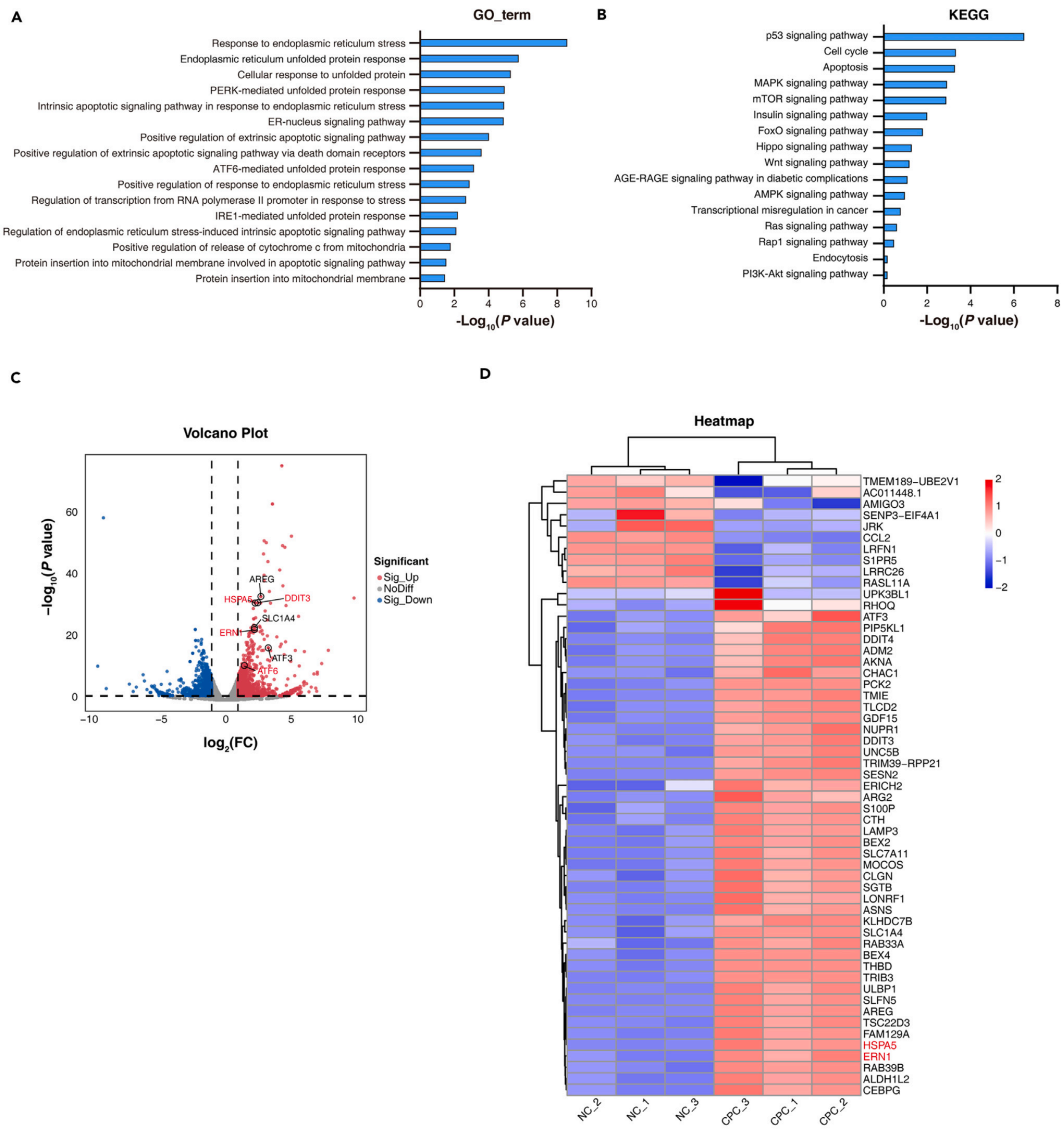
CPC alters ER-related pathways in pancreatic cancer (A) Gene ontology (GO) analysis of biological processes with differentially expressed genes from RNA sequencing data in normal control (NC) and CPC (7.5 μM) treatment for 24 h in PANC1 cells. (B) Kyoto Encyclopedia of Genes and Genomes (KEGG) pathway enrichment analysis with differentially expressed genes from RNA sequencing data in NC group and CPC treatment group in PANC1 cells. (C) Volcano plots represent the differentially expressed genes from RNA sequencing data in the NC group and CPC treatment group in PANC1 cells. (D) Heatmap representation of differentially expressed genes between NC group and CPC treatment group from RNA sequencing data (*n* = 3 biologically independent samples).

To explore the molecular mechanism driving CPC-induced cell death, we conducted RNA sequencing on PANC1 cells treated with 7.5 μM CPC for 24 h. This allowed for a comparative analysis between the negative control group and the CPC-treated group. Our gene ontology (GO) analysis of biological processes revealed that CPC significantly influenced the ER stress response, unfolded protein response, and ER nuclear signaling pathways (Figure 4A). These findings align with the observed alterations in ER morphology caused by CPC.

Pathway enrichment analysis of differentially expressed genes revealed that CPC had a significant impact on the tumor protein p53 (TP53) signaling pathway (Figure 4B), regulating multiple cell death pathways, including apoptosis and paraptosis.^35^ While CPC-induced apoptosis was not observed, pathway enrichment analysis also indicated apoptosis as a potential outcome (Figure 4B), given that several regulators, such as TP53, govern apoptosis, autophagy, and paraptosis.^36^ Furthermore, our volcano and heatmap analyses showed that CPC upregulated ER stress-related genes (ERN1, heat shock protein family A [Hsp70] member 5 [HSPA5, also known as BIP], DNA damage-inducible transcript 3 [DDIT3, also known as CHOP], and activating transcription factor 6 [ATF6]) in PDAC cells) (Figures 4C and 4D). These results collectively suggest that CPC alters ER-related biological processes and pathways, leading to the upregulation of ER stress-related genes.

**Figure 5:**
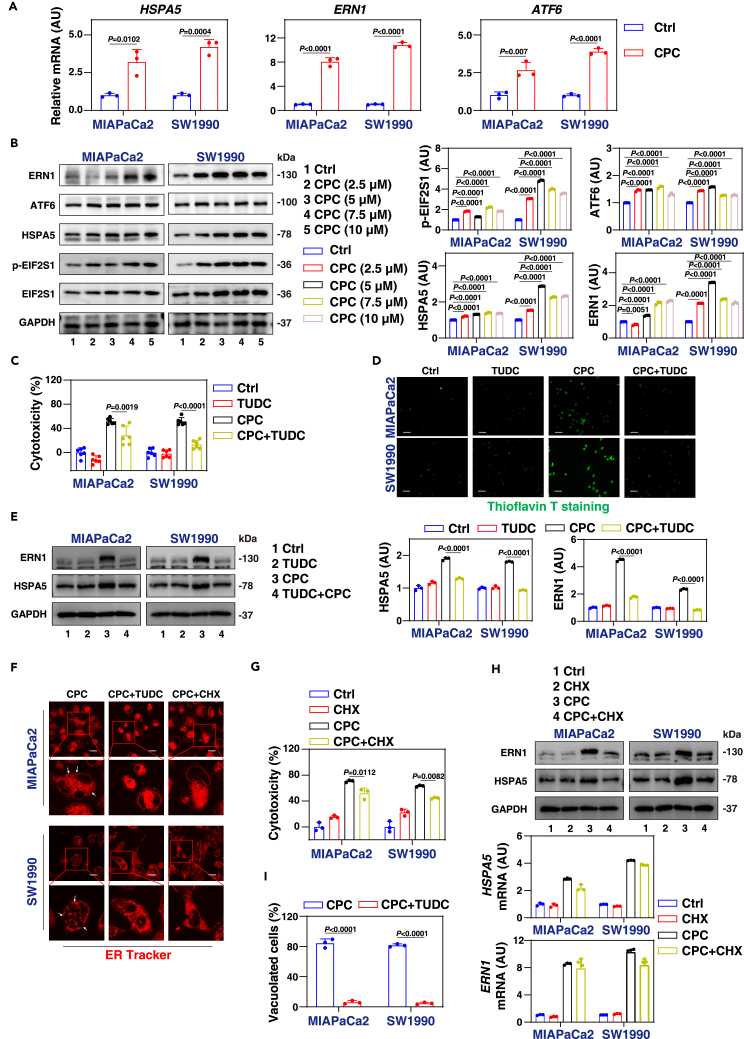
CPC induces ER stress and paraptosis in pancreatic cancer (A) A qPCR analysis of the mRNA expression of indicated genes in MIAPaCa2 and SW1990 cells following treatment with CPC (7.5 μM) for 24 h. (B) Western blot analysis of the expression of indicated proteins in MIAPaCa2 and SW1990 cells after treatment with CPC for 24 h. (C) Cytotoxicity of MIAPaCa2 and SW1990 cells after treatment with CPC (7.5 μM) in the absence or presence of sodium tauroursodeoxycholate (TUDC; 1 mM) for 24 h. (D) ER stress-related amyloid deposition was examined using the fluorescent stain thioflavin-T (green color). Scale bar: 200 μm. (E) Western blot analysis of protein expression of MIAPaCa2 and SW1990 cells after treatment with CPC (7.5 μM) in the absence or presence of TUDC (1 mM) for 24 h. (F) MIAPaCa2 and SW1990 cells were treated with CPC (7.5 μM) in the absence or presence of cycloheximide (CHX; 5 μM) or TUDC (1 mM) for 24 h. ER structures were stained with ER-Tracker Red. Scale bar: 10 μm. (G) Cytotoxicity of MIAPaCa2 and SW1990 cells after treatment with CPC (7.5 μM) in the absence or presence of CHX (5 μM) for 24 h. (H) Analysis of protein and gene expression in MIAPaCa2 and SW1990 cells after treatment with CPC (7.5 μM) in the absence or presence of CHX (5 μM) for 24 h. (I) Light microscopy analysis of cell morphology in MIAPaCa2 and SW1990 cells after treatment with CPC (7.5 μM) in the absence or presence of TUDC (1 mM) for 24 h. Scale bar: 50 μm. Data are presented as mean ± SD. The proportion of cells displaying vacuoles was determined by visually examining a minimum of 100 cells and the data are presented as mean ± SD from three independent experiments (I). Statistical significance was assessed using one-way ANOVA with Dunnett’s multiple comparisons test (B, C, E, G, and H) or unpaired two-tailed Student’s t tests (A and I). See also Figures S3.

Considering CPC’s capacity to induce ER morphological dilation and the results of RNA sequencing, we further investigated whether CPC initiates ER stress in pancreatic cancer cells. ER stress activates the unfolded protein response, an adaptive mechanism that reduces the burden of unfolded proteins to maintain cellular function.^37^ Quantitative polymerase chain reaction (qPCR) and western blot analysis revealed that CPC treatment upregulates the expression of HSPA5, ERN1, and ATF6 (Figures 5A and 5B), or induces the phosphorylation of eukaryotic translation initiation factor 2 subunit alpha (EIF2S1; Figure 5B), all of which are well-known markers associated with ER stress and the unfolded protein response.^38^

To confirm the involvement of ER stress in CPC-induced cell death, we conducted pretreatment experiments with the ER stress inhibitor sodium tauroursodeoxycholate (TUDC).^39^ TUDC rescued CPC-induced cell death (Figure 5C), indicating that ER stress mediates CPC-induced cell death rather than providing cellular protection. Additionally, thioflavin T staining, which marks ER stress and intracellular misfolded protein aggregation,^40^ showed that CPC treatment resulted in the accumulation of misfolded protein aggregation in cells. In contrast, pretreatment with TUDC reduced this accumulation (Figure 5D). Consistently, pretreatment with TUDC attenuated the upregulation of ERN1 and HSPA5 protein expression induced by CPC, further supporting the attenuation of CPC-induced ER stress (Figure 5E).

Paraptosis is a non-apoptotic form of cell death characterized by ER stress, extensive ER-derived cytoplasmic vacuolization, and eventual plasma membrane rupture.^41^ To further investigate whether CPC induces paraptosis, we pretreated MIAPaCa2 and SW1990 cells with CHX, a protein synthesis inhibitor known to inhibit paraptosis.^42^ CHX pretreatment reduced CPC-induced ER dilatation (Figure 5F) and inhibited the anticancer activity of CPC (Figure 5G). Furthermore, CHX pretreatment prevented CPC-induced upregulation of ER stress proteins (ERN1 and HSPA5; Figure 5H), but did not alter the mRNA expression levels of the genes encoding these proteins (Figure 5H). Pretreatment with TUDC also blocked CPC-induced vacuolization and ER dilatation (Figures 5F, 5I, and S3A).

Collectively, these findings indicate that CPC induces ER stress, leading to subsequent paraptosis.

### ERN1 is a positive regulator of paraptosis

**Figure 6:**
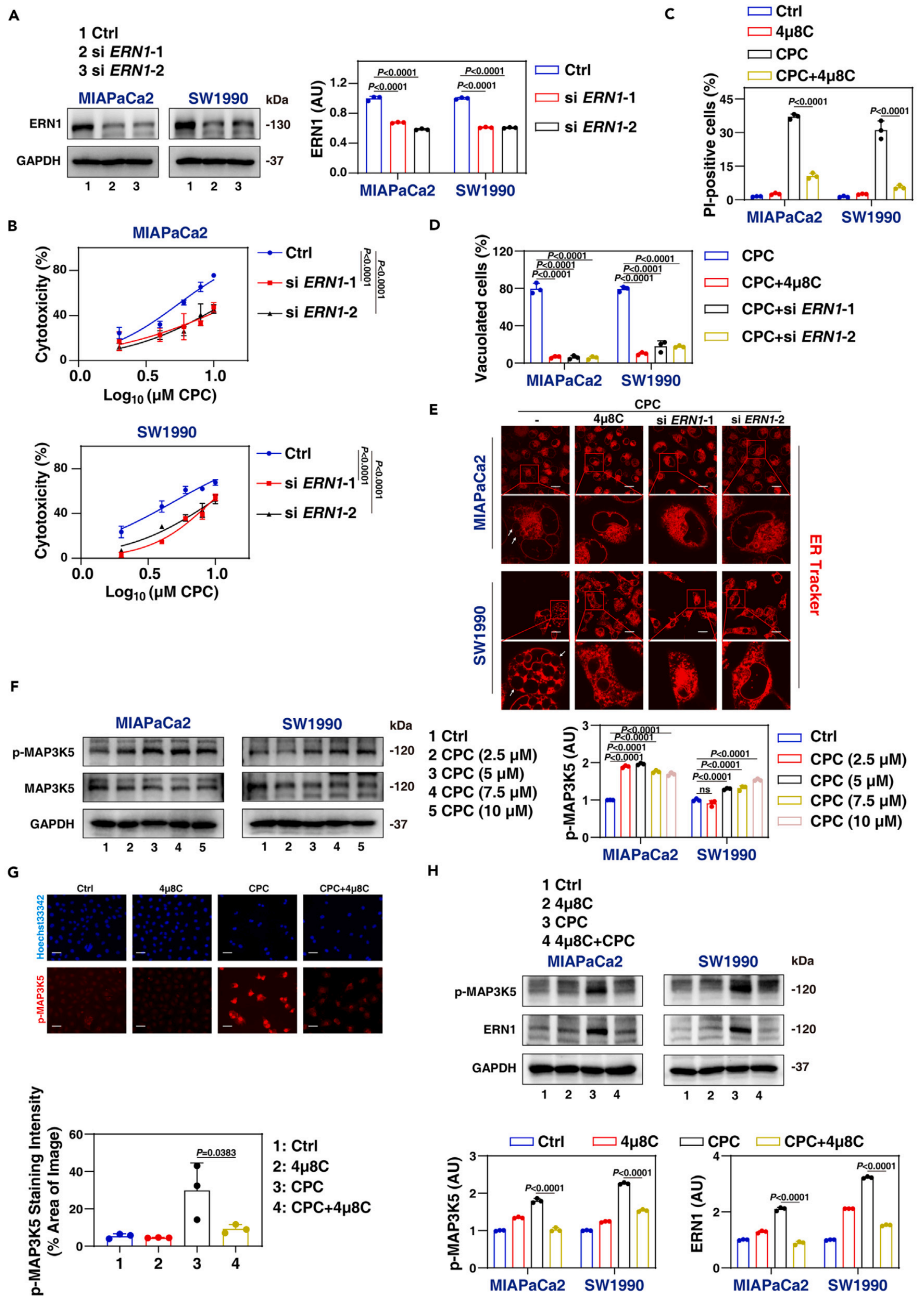
ERN1 mediates paraptosis and activates MAP3K5 (A) Western blot analysis of protein expression in control and *ERN1*-knockdown PDAC cells. (B) The cytotoxicity of indicated MIAPaCa2 and SW1990 cells after treatment with CPC for 24 h. (C) PI staining of cell death in MIAPaCa2 and SW1990 cells after treatment with CPC (7.5 μM) in the absence or presence of 4μ8C (100 μM) for 24 h. (D) Light microscopy analysis of cell morphology in indicated MIAPaCa2 and SW1990 cells after treatment with CPC (7.5 μM) in the absence or presence of 4μ8C (100 μM) for 24 h, followed by quantitative assessment. (E) Indicated MIAPaCa2 and SW1990 cells were treated with CPC (7.5 μM) in the absence or presence of 4μ8C (100 μM) for 24 h. ER structures were stained with ER-Tracker Red. Scale bar: 10 μm. (F) Western blot analysis of protein expression of indicated MIAPaCa2 and SW1990 cells after treatment with CPC for 24 h. (G) MIAPaCa2 and SW1990 cells were treated with CPC (7.5 μM) in the absence or presence of 4μ8C (100 μM) for 24 h. Cells were stained with Hoechst 33342 (blue) and p-MAP3K5 antibody (red), followed by quantitative assessment. (H) Western blot analysis of protein expression in MIAPaCa2 and SW1990 cells following treatment with CPC (7.5 μM) in the absence or presence of 4μ8C (100 μM) for 24 h. Data are presented as mean ± SD. The proportion of cells displaying vacuoles was determined by visually examining a minimum of 100 cells, and the data are presented as the mean ± SD from at least three independent experiments (D). *p* values were calculated using one-way ANOVA with Dunnett’s multiple comparisons test (A, C, D, F, G, and H) or two-way ANOVA (B). See also Figures S3.

To identify the specific ER stress sensor involved in CPC-induced paraptosis, we individually knocked down three ER transmembrane pressure sensors: ERN1, ATF6, and eukaryotic translation initiation factor 2 alpha kinase 3 (EIF2AK3, also known as PERK), using specific siRNAs (Figures 6A and S3B). The knockdown of *EIF2AK3* or *ATF6* did not rescue CPC-induced cytotoxicity, while the knockdown of *ERN1* inhibited CPC-induced cytotoxicity (Figure 6B and S3C). Moreover, pretreatment with the ERN1 inhibitor 4μ8C blocked CPC-induced cell death and cytotoxicity (Figures 6C, S3D, and S3E). Consistently, *ERN1* knockdown or pretreatment with 4μ8C attenuated CPC-induced vacuolization and ER dilatation in both MIAPaCa2 and SW1990 cells (Figures 6D, 6E, and S3F). These genetic and pharmacological experiments confirm that ERN1 plays a major role in mediating CPC-induced cell death.

Activation of the MAPK signaling pathway is another hallmark of paraptotic cell death initiated by ER stress.^20^ Pathway enrichment analyses using RNA sequencing also suggest the involvement of the MAPK pathway in CPC-induced cell death (Figure 4B). Previous studies have suggested that either MAP3K5 (also known as ASK1) or X-box binding protein 1 (XBP1) can regulate MAPK signaling downstream of ERN1.^43^^,^^44^ We observed an increase in MAP3K5 phosphorylation while XBP1 did not undergo splicing in response to CPC treatment (Figures 6F and S3G). As a positive control,^45^ thapsigargin, a classical ER stress inducer, induced XBP1 splicing (Figure S3H). Western blot and immunofluorescence assays further demonstrated that 4μ8C inhibited MAP3K5 phosphorylation (Figures 6G and 6H).

Taken together, these findings suggest that MAP3K5 phosphorylation, rather than XBP1 splicing, is involved in the activation of the ERN1 pathway in response to CPC treatment.

### Activation of p38 drives CPC-induced paraptosis

**Figure 7:**
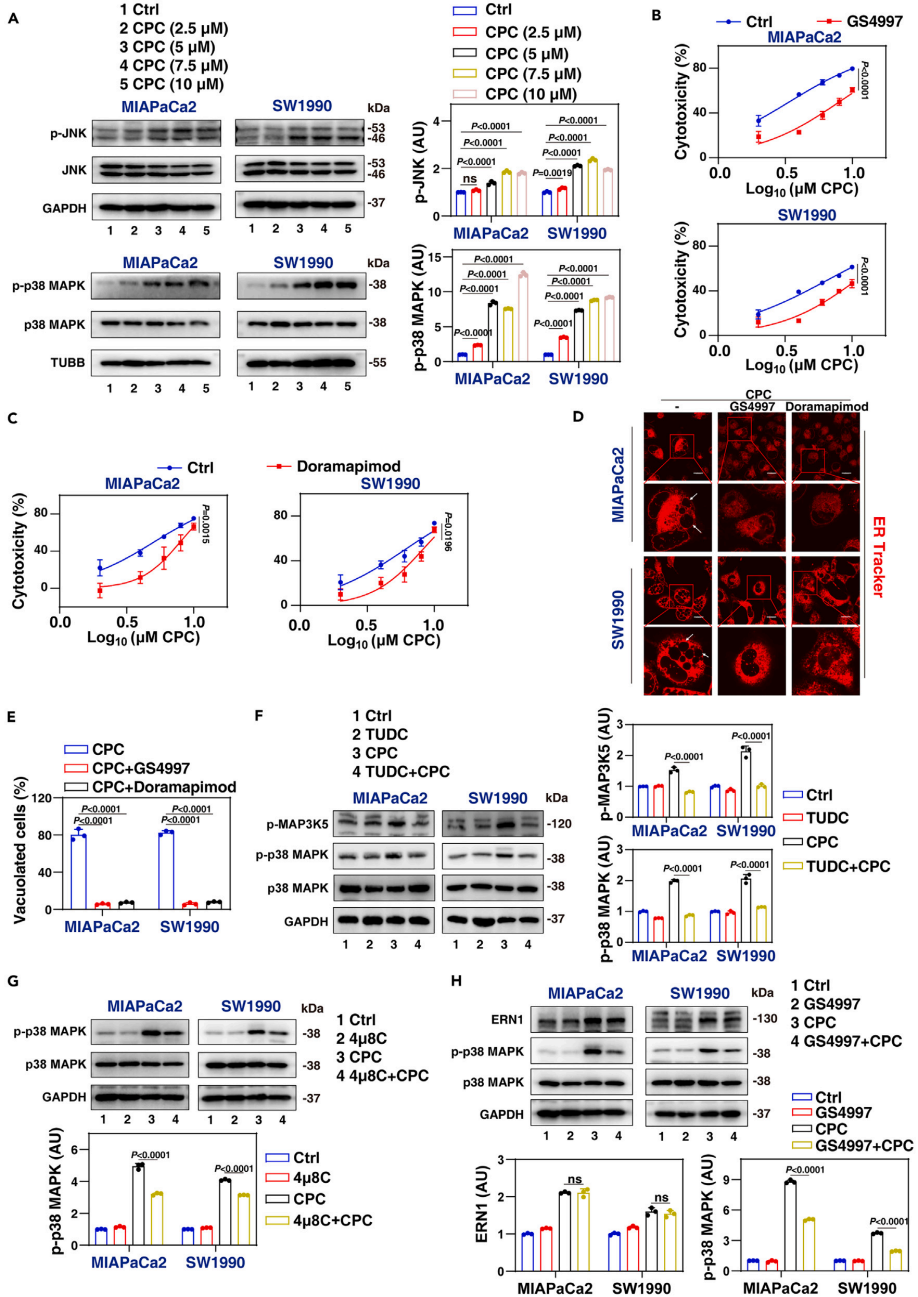
Evidence that p38 is critical for CPC-induced paraptosis (A) Western blot analysis of protein expression in MIAPaCa2 and SW1990 cells after treatment with CPC for 24 h. (B and C) Cytotoxicity of MIAPaCa2 and SW1990 cells treated with CPC in the absence or presence of the GS4997 (10 μM) or doramapimod (5 μM) for 24 h. (D) MIAPaCa2 and SW1990 cells were treated with CPC (7.5 μM) in the absence or presence of GS4997 (10 μM) or doramapimod (5 μM) for 24 h. ER structures were stained with ER-Tracker Red. Scale bar: 10 μm. (E) Light microscopy analysis of cell morphology in indicated MIAPaCa2 and SW1990 cells after treatment with CPC (7.5 μM) in the absence or presence of GS4997 (10 μM) or doramapimod (5 μM) for 24 h, followed by quantitative assessment. (F–H) Western blot analysis of protein expression in MIAPaCa2 and SW1990 cells after treatment with CPC (7.5 μM) in the absence or presence of tauroursodeoxycholate (TUDC; 1 mM), 4μ8C (100 μM), or GS4997 (10 μM) for 24 h. Data are presented as mean ± SD. The proportion of cells displaying vacuoles was determined by visually examining a minimum of 100 cells, and the data are presented as the mean ± SD from at least three independent experiments (E). *p* values were calculated by one-way ANOVA (A, E, F, G, and H) or two-way ANOVA (B and C). See also Figure S4.

Given that MAP3K5 is a protein kinase known to activate the MAPK pathways, including p38 and c-Jun N-terminal kinases (JNK),^46^ we further investigated the impact of CPC on the phosphorylation of p38 and JNK. Western blot analysis revealed an increase in the phosphorylation levels of p38 MAPK and JNK in CPC-treated pancreatic cancer cells (Figure 7A).

To explore the association between MAP3K5, p38, JNK, and CPC-induced paraptosis, we utilized specific inhibitors: GS4997 (a MAP3K5 inhibitor), doramapimod (a p38 MAPK inhibitor), and SP600125 (a JNK inhibitor). CCK-8 assays revealed that both doramapimod and GS4997 inhibited CPC-induced cytotoxicity (Figures 7B, 7C, and S4A). However, SP600125 failed to inhibit CPC cytotoxicity although it successfully inhibited H_2_O_2_-induced cytotoxicity as a positive control (Figure S4B).

Furthermore, pretreatment with doramapimod or GS4997 reduced the formation of CPC-induced vacuoles and ER dilatation (Figures 7D, 7E, and S4C). Additionally, both TUDC and CHX inhibited CPC-induced activation of p38 MAPK (Figures 7F and S4D), demonstrating that ER stress indeed serves as an upstream signal for triggering MAPK activation. Likewise, the ERN1 inhibitor 4μ8C and the MAP3K5 inhibitor GS4997 also inhibited p38 MAPK activation (Figures 7G and 7H). In contrast, the addition of the p38 inhibitor doramapimod did not affect CPC-induced upregulation of ERN1 expression (Figure S4E), supporting the hypothesis that p38 acts as a downstream signal of ERN1 activation.

To determine whether CPC specifically initiates paraptosis in PDAC cells, we subjected OVCAR-3 cells (human ovarian cancer) and HeLa cells (human cervical cancer) to CPC treatments. CPC induced cytotoxicity in both OVCAR-3 and HeLa cells in a dose-dependent manner (Figure S4F). However, neither vacuole formation nor activation of the ERN1-MAP3K5-p38 MAPK signaling cascade pathway was observed in OVCAR-3 or HeLa cells treated with CPC (Figures S4G and S4H).

These findings collectively suggest that the activation of the ERN1-MAP3K5-p38 pathway triggered by ER stress plays a pivotal role in CPC-induced paraptosis in PDAC cells.

### CPC suppresses pancreatic cancer tumor growth *in vivo*

**Figure 8:**
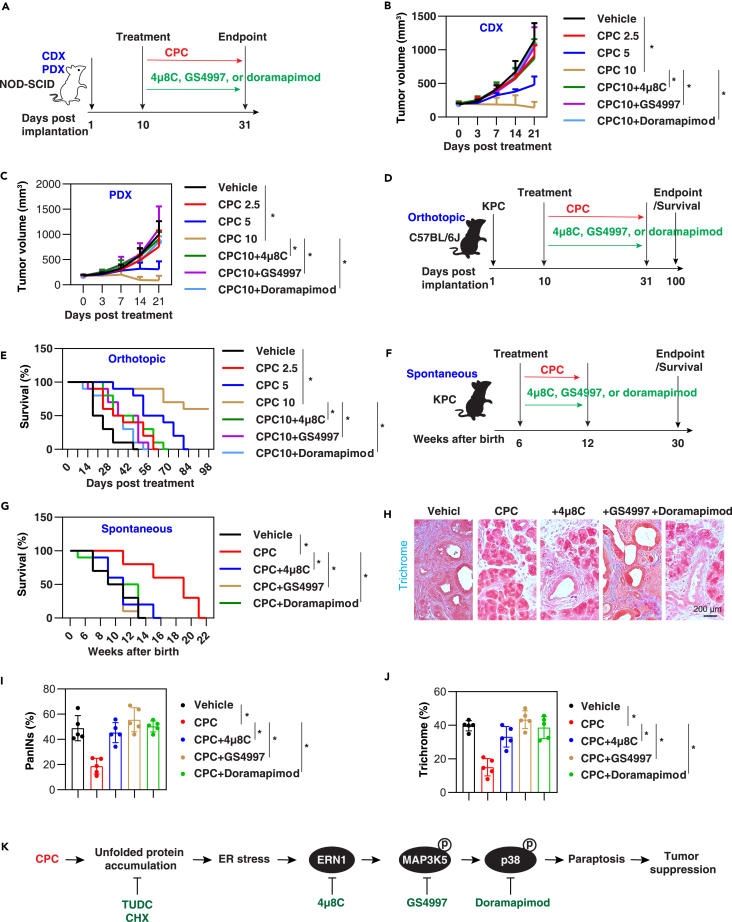
CPC suppresses pancreatic cancer tumor growth *in vivo* (A) A schematic diagram illustrating the experimental setup involving the subcutaneous implantation of human PANC1 cells (CDXs) or PDXs in immunocompromised NSG-SCID mice, followed by a 3-week treatment protocol. During this period, the mice received either a vehicle or CPC (10 mg/kg orally, once daily, 5 days per week), under conditions with or without 4μ8C (20 mg/kg orally, once daily, 5 days per week), GS4997 (10 mg/kg orally, once daily, 5 days per week), or doramapimod (20 mg/kg orally, once daily, 5 days per week). CDX models involve the transplantation of established cancer cell lines into mice. PDX models involve the transplantation of cancerous tissue or isolated cells from a patient into immunocompromised mice. (B and C) Tumor growth curves of CDXs or PDXs implanted subcutaneously into NSG-SCID mice (*n* = 5 mice per group). (D) A schematic diagram illustrating the experimental setup involving the orthotopic implantation of KPC cells in C57BL6/J mice, followed by a 3-week treatment protocol. During this period, the mice received either a vehicle or CPC (10 mg/kg orally, once daily, 5 days per week), under conditions with or without 4μ8C (20 mg/kg orally, once daily, 5 days per week), GS4997 (10 mg/kg orally, once daily, 5 days per week), or doramapimod (20 mg/kg orally, once daily, 5 days per week). An orthotopic model of pancreatic cancer is a specific type of animal model where pancreatic cancer cells or tissues are surgically implanted into the pancreas of the host animal. (E) Survival curves of indicated mice treated with vehicle, CPC, 4μ8C, GS4997, and/or doramapimod (*n* = 10 mice/group). (F) A schematic diagram illustrating the experimental setup involving the KPC mice followed by a 6-week treatment protocol. During this period, the mice received either a vehicle or CPC (10 mg/kg orally, once daily, 5 days per week), under conditions with or without 4μ8C (20 mg/kg orally, once daily, 5 days per week), GS4997 (10 mg/kg orally, once daily, 5 days per week), or doramapimod (20 mg/kg orally, once daily, 5 days per week). (G) Survival curves of indicated mice treated with vehicle, CPC, 4μ8C, GS4997, and/or doramapimod (*n* = 10 mice/group). (H–J) Pancreatic sections from KPC mice, following 4 weeks of treatment with vehicle, CPC, 4μ8C, GS4997, and/or doramapimod, were subjected to trichrome staining to visualize both the structural changes and stromal response. Quantification of relative expression was performed (*n* = 5 mice/group) using one-way ANOVA with Tukey’s multiple comparisons test. The results are presented as mean ± SD. (K) A schematic diagram illustrating how CPC-induced paraptosis contributes to the eradication of pancreatic cancer via the ERN1-MAP3K5-p38 pathway.

To assess the potential application of CPC in treating PDAC, we initially employed human xenograft models. PANC1 cells were subcutaneously injected into immunocompromised non-obese diabetic-severe combined immunodeficiency (NOD-SCID) mice (Figure 8A). Oral administration of CPC (2.5–10 mg/kg) produced a dose-dependent inhibition of tumor growth in the PANC1 cell line-derived xenografts (CDXs; Figure 8B). This effect was subsequently reversed by the administration of the ERN1 inhibitor 4μ8C, the MAP3K5 inhibitor GS4997, or the p38 inhibitor doramapimod (Figure 8B). Similarly, PDAC patient-derived tumor xenograft (PDX) models in NOD-SCID mice also confirmed that CPC-induced tumor suppression relies on the ERN1-MAP3K5-p38 pathway (Figures 8A and 8C).

Next, we examined whether the anticancer activity of CPC is also effective in immune-intact mice. We injected KPC cells (derived from pancreatic tumors of *K-Ras*^*G12D*^*;Tp53*^*R172H*^*;Pdx1-Cre* mice) into the pancreas of C57BL/6J mice with orthotopic models and observed that CPC prolonged the survival (Figures 8D and 8E). This protective effect of CPC on animal survival was also reversed by the administration of the ERN1 inhibitor 4μ8C, the MAP3K5 inhibitor GS4997, or the p38 inhibitor doramapimod (Figure 8E).

Finally, we used transgenic spontaneous PDAC models, namely KPC mice, which are aggressive PDAC models with an average animal survival of around 2–3 months (Figures 8F and 8G). The administration of CPC at the age of 6 weeks, followed by twice-weekly treatments, doubled the median survival time of KPC mice (Figure 8G). Trichrome staining revealed that CPC reduced the proportion of pancreatic intraepithelial neoplasia (PanIN) and stromal response in KPC mice at 10 weeks after birth (Figures 8H–8J). However, the animal survival and histological protection effects of CPC were reversed by the ERN1 inhibitor 4μ8C, the MAP3K5 inhibitor GS4997, and the p38 inhibitor doramapimod (Figures 8G and 8H–8J).

Altogether, these diverse mouse tumor models provide *in vivo* evidence that CPC effectively inhibits pancreatic tumor growth through the ERN1-MAP3K5-p38 pathway.

## Discussion

PDAC is the most prevalent form of pancreatic malignancy.^47^ PDAC has the highest mortality rate among major cancers, and while current standard treatment involves cytotoxic chemotherapy (e.g., nab-paclitaxel plus gemcitabine and FOLFIRINOX) combined with supportive therapy, the need for more effective options remains critical.^48^ In this preclinical study, we present evidence supporting the potential of CPC to trigger paraptosis, thereby effectively suppressing and inhibiting PDAC *in vitro* and in three clinically relevant mouse models (Figure 8K). CPC is a common active ingredient in antiseptic oral mouth rinses. Therefore, our findings present a promising opportunity for repurposing CPC in PDAC treatment. This study also yields valuable insights that can facilitate further clinical research using CPC in tumor therapy.

To identify potential drugs for PDAC, we conducted a screening of FDA-approved drugs, leading to the discovery of CPC as a candidate, which currently has limited reports in cancer treatment. Our screening results revealed an approximate IC50 value of ∼5 μM in various PDAC cell lines. Exploring the chemical structure underlying CPC-induced cell death and strategies to increase the IC50 to nanomolar levels would be intriguing directions for future research. Moreover, our present study provides evidence of CPC’s direct induction of paraptosis in PDAC cells, while research from other laboratories suggests that CPC may have the capacity to influence the gut microbiome and modulate antitumor immune responses.^49^^,^^50^^,^^51^

The induction of cancer cell death represents a fundamental objective in tumor therapy.^52^^,^^53^ Traditional cytotoxic agents typically initiate apoptosis, a process driven by caspase activation leading to the cleavage of various substrates, including PARP1, which is among the earliest identified protein substrates in apoptotic activation.^54^ While we observed PARP1 cleavage in response to CPC treatment, we did not detect CASP3 cleavage. In addition, the pan-caspase inhibitor Z-VAD-FMK did not inhibit the production of cleaved PARP1 during CPC treatment, suggesting that CPC-induced PARP cleavage occurs through a non-caspase pathway. Other proteases, such as cathepsins, calpains, matrix metalloproteinases, and granzymes, can also cleave PARP1 under different experimental conditions.^55^^,^^56^^,^^57^^,^^58^ Further investigations are required to ascertain whether these proteases directly mediate PARP1 cleavage following CPC treatment.

Our findings highlight that excessive ER stress signals trigger paraptosis, characterized by the formation of cytoplasmic vacuoles. While the term “paraptosis” was first coined in 2000 to investigate the mechanism of insulin-like growth factor 1 receptor (IGF-1R)-induced cell death,^59^ the molecular mechanism and regulation of paraptosis remain incompletely understood. Since then, several small-molecule compounds have been shown to induce paraptosis in cancer cells, which cannot be inhibited by apoptosis inhibitors.^60^ Morphologically, paraptotic cells display distinct cytoplasmic vacuole formation and swelling of the ER and mitochondria.^59^ At the molecular level, classical regulators of the mitochondrial pathway, such as caspases and BCL2 family members, are not pivotal in regulating paraptosis. Once the number and size of cytoplasmic vacuoles surpass a certain threshold, paraptotic cell death becomes irreversible. Moreover, both pharmacological and genetic interventions targeting autophagy do not affect the formation of CPC-induced cytoplasmic vacuoles, suggesting that the induction of autophagy may represent an associated phenotype rather than a causal factor in the formation of CPC-induced cytoplasmic vacuoles.

While paraptosis is commonly associated with ER stress and subsequently the unfolded protein response,^61^ our experimental model revealed that among the three ER stress sensors (ERN1, ATF6, and EIF2AK3), only ERN1 is involved in the initiation of paraptosis. This observation suggests that different ER stress signals may elicit distinct modes of cell death. We emphasize the significance of the activation of the ERN1-MAP3K5-p38 signaling pathway as a major contributor to CPC-induced vacuole formation and paraptosis as well as tumor suppression across multiple PDAC mouse models. Although individual components of this signaling pathway have been reported to regulate apoptosis,^62^^,^^63^ it seems that the entire pathway is accountable for CPC-induced paraptosis in PDAC cells, rather than apoptosis. Nevertheless, other unknown factors, such as genetic mutations in different cancer types, may impact CPC activity. While we observed CPC’s cytotoxic effects on HeLa and OVCAR-3 cells, it did not activate the ERN1-p38 pathway or induce paraptosis. This observation may be linked to the presence of KRAS mutations commonly found in PDAC. Oncogenic KRAS mutations have been reported to stabilize ERN1 by regulating its ubiquitination level,^64^ potentially rendering ERN1 more prone to activation in the context of protein homeostasis dysregulation. Additionally, the p38 MAPK pathway plays a pivotal role in transmitting stress signals from the extracellular environment, and KRAS mutations can lead to aberrant activation of p38 MAPK.^65^ Therefore, assessing the activation of the entire pathway along with the genetic background of various tumor types is crucial for monitoring CPC activity in mediating distinct forms of cell death.

In cells, p38 MAPK acts as a signal transduction hub and plays a complex role in cell growth and death, depending on the upstream signals and downstream effectors involved.^66^ In tumors, an excessive activation of p38 MAPK has been associated with malignancy and drug resistance.^67^ Nevertheless, our *in vitro* and *in vivo* investigations demonstrate that p38 kinase activation is a prerequisite for CPC-induced paraptosis and subsequent tumor suppression. Further elucidation of p38 substrate proteins may unveil the effectors responsible for CPC-induced cell death. Irrespective of the mechanism, CPC exhibits the potential for selectively targeting p38-positive cancers, including PDAC, which often exhibit resistance to conventional chemotherapy.^68^

In summary, we highlight the potential of CPC for PDAC treatment through ER stress-induced paraptotic cell death. These findings broaden our understanding of ER stress and MAPK signaling in cell death and may present avenues for pancreatic cancer treatment.

### Limitations of the study

Although our study demonstrated that CPC selectively induces paraptosis to inhibit pancreatic cancer growth, further research is necessary to evaluate its long-term efficacy, safety profile, and pharmacokinetics *in vivo*. This includes detailed assessment of its concentration and metabolism across various tissues, particularly the pancreas. Additionally, understanding how CPC induces apoptosis and paraptosis in various tumor types remains unclear. Moreover, the direct protein target of CPC in cell death pathways requires further identification. In the antimicrobial mechanism, CPC has been demonstrated to bind to proteins on the cell membrane, causing cell content leakage and dysfunction of membrane-associated proteins.^9^ Further investigation is needed to determine whether CPC exerts its anti-tumor activity through similar mechanisms involving plasma membrane proteins.

## STAR★Methods

### Key resources table

**Table:** 

REAGENT or RESOURCE	SOURCE	IDENTIFIER
**Antibodies**		

Goat anti-mouse IgG-HRP	Beyotime	Cat# A0216; RRID: AB_2860575
Goat anti-rabbit IgG-HRP	Beyotime	Cat# A0208; RRID: AB_2892644
Mouse monoclonal anti-HSPA5	Proteintech Biotechnology	Cat# 66574-1-Ig; RRID: AB_2881934
Rabbit polyclonal anti-ACTB	Affinity Biosciences	Cat# AF7018; RRID: AB_2839420
Rabbit polyclonal anti-ATF6	Proteintech Biotechnology	Cat# 24169-1-AP; RRID: AB_2876891
Rabbit polyclonal anti-CASP3	Proteintech Biotechnology	Cat# 19677-1-AP; RRID: AB_10733244
Rabbit polyclonal anti-EIF2AK3	Proteintech Biotechnology	Cat# 24390-1-AP; RRID: AB_2879521
Rabbit polyclonal anti-EIF2S1	Proteintech Biotechnology	Cat# 11170-1-AP; RRID: AB_2096489
Rabbit polyclonal anti-ERN1	Proteintech Biotechnology	Cat# 27528-1-AP; RRID: AB_2880899
Rabbit polyclonal anti-GAPDH	Affinity Biosciences	Cat# 7021; RRID: AB_2839421
Rabbit polyclonal anti-GPX4	Affinity Biosciences	Cat#DF6701; RRID: AB_2838663
Rabbit polyclonal anti-p38 MAPK	Cell Signaling Technology	Cat# 9212; RRID: AB_2861335
Rabbit polyclonal anti-PARP1	Proteintech Biotechnology	Cat# 13371-1-AP; RRID: AB_2160459
Rabbit polyclonal anti-*p*-EIF2S1	Bimake	Cat# A5941; RRID: AB_2834524
Rabbit polyclonal anti-p-MAP3K5	Affinity Biosciences	Cat# AF8096; RRID: AB_2840159
Rabbit polyclonal anti-MLKL	Affinity Biosciences	Cat# DF7412; RRID: AB_2839350
Rabbit polyclonal anti-*p*-MLKL	Affinity Biosciences	Cat# AF7420; RRID: AB_2843860
Rabbit polyclonal anti-p-p38 MAPK (Thr180/Tyr182)	Cell Signaling Technology	Cat# 9211; RRID: AB_2918205
Rabbit polyclonal anti-TUBB	Affinity Biosciences	Cat# AF8096; RRID: AB_2827688
Rabbit polyclonal anti-XBP1s	Proteintech Biotechnology	Cat# 24868-1-AP; RRID: AB_2879766
Rabbit recombinant anti-*p*-JNK (Tyr185)	Proteintech Biotechnology	Cat# 80024-1-RR; RRID: AB_2882943
Rabbit recombinant polyclonal anti-JNK	Bimake	Cat# A5005; RRID: AB_2266214
Rabbit recombinant polyclonal anti-MAP3K5	Bimake	Cat# A5432; RRID: AB_2782957
Rabbit recombinant polyclonal anti-MAPLC3B	Bimake	Cat# A5202; RRID: AB_2137737
Rabbit recombinant polyclonal anti-SQSTM1	Bimake	Cat# A5180; RRID: AB_10694431
Rabbit recombinant polyclonal anti-XBP1u	Bimake	Cat# A5763; RRID: AB_2879445
Rabbit recombinant polyclonal anti-ATG5	Proteintech Biotechnology	Cat# 10181-2-AP; RRID: AB_2062045
Rabbit recombinant polyclonal anti-ATG7	Proteintech Biotechnology	Cat# 10088-2-AP; RRID: AB_2062351

**Biological samples**		

Fetal bovine serum	Thermo Fisher Scientific	Cat# A3840001

**Chemicals, peptides, and recombinant proteins**		

3-methyladenine	Selleck Chemicals	Cat# S2767
4μ8C	Selleck Chemicals	Cat# S7272
BCA	Thermo Fisher Scientific	Cat# 23225
Cell lysis buffer	Biosharp	Cat# BL509A
Cetylpyridinium chloride	Selleck Chemicals	Cat# S4172
Chloroquine	Selleck Chemicals	Cat# S6999
Crystal violet	Solarbio Life Science	Cat# C8470
Cycloheximide	Selleck Chemicals	Cat# S7418
Doramapimod	Selleck Chemicals	Cat# S1574
Dulbecco’s modified Eagle’s medium	Thermo Fisher Scientific	Cat# 11995073
ER-Tracker Red	Beyotime	Cat# C1041S
Ferrostatin-1	Selleck Chemicals	Cat# S7243
GS4997	Selleck Chemicals	Cat# S8292
Lipofectamine RNA iMAX	ThermoFisher Scientific	Cat# 13778500
LysoTracker Green	Beyotime	Cat# C1047S
MitoTracker Green	Beyotime	Cat# C1048
Necrosulfonamide	Selleck Chemicals	Cat# S8251
Opti-MEM I	Gibco	Cat# 31985070
Polyacrylamide gel electrophoresis gels	Epizyme	Cat# PG112
Polyvinylidene difluoride membranes	Millipore	Cat# IPVH00010
Protease and phosphatase inhibitor cocktail	Cell Signaling Technology	Cat# 5872
Protease inhibitor cocktail	Cell Signaling Technology	Cat# 5872
Puromycin	YEASEN	Cat# 60210ES72
RSL3	Selleck Chemicals	Cat# S8155
Sodium tauroursodeoxycholate	Selleck Chemicals	Cat# S7896
SP600125	Selleck Chemicals	Cat# S1460
Staurosporine	Selleck Chemicals	Cat# S1412
Streptomycin/penicillin	Yeasen	Cat# 60162ES76
TB Green Premix Ex Taq II	Takara	Cat# RR820Q
Thapsigargin	Selleck Chemicals	Cat# S7895
Thioflavin T	Selleck Chemicals	Cat# S6873
ZVAD-FMK	Selleck Chemicals	Cat# S7023
Rapamycin	Selleck Chemicals	Cat# S1039

**Critical commercial assays**		

Cell Counting Kit-8	YEASEN	Cat# 40203ES80
Hoechst 33342 and propidium iodide	BestBio	Cat# BB-4131-1
Plus Micro Kit	QIAGEN	Cat# 74034
PrimeScript RT Master Mix	Takara	Cat# RR036A

**Deposited data**		

The RNA-seq data	Sequence Read Archive	PRJNA1040747

**Experimental models: cell lines**		

293FT cells	Thermo Fisher Scientific	Cat# R70007
Human PDAC cell line MIAPaCa2	American Type Culture Collection	Cat# CRL-1420
Human PDAC cell line PANC1	American Type Culture Collection	Cat# CRL-1469
Human PDAC cell line SW1990	American Type Culture Collection	Cat# CRL-2172
Human cervical cancer cell line Hela	American Type Culture Collection	Cat# CRM-Ccl2
Human ovarian carcinoma cell line OVCAR-3	American Type Culture Collection	Cat# HTB-161

**Experimental models: organisms/strains**		

C57BL/6J mice	The Jackson Laboratory	Cat# 000664
KPC mice	David Tuveson	N/A
NOD-SCID mice	The Jackson Laboratory	Cat# 001303

**Oligonucleotides**		

Primers for various genes	This paper	STAR Methods section

**Recombinant DNA**		

Various genes	This paper	STAR Methods section

**Software and algorithms**		

Bio-Rad CFX Manager software 2.0	Bio-Rad CFX Manager	https://www.bio-rad.com/en-hk/sku/1845000-cfx-manager-software?ID=1845000
GraphPad Prism 8.02	GraphPad	https://www.graphpad.com/
ImageJ	ImageJ	https://imagej.net/software/imagej/
Image Lab 5.2.1	Image Lab	https://www.bio-rad.com/en-hk/product/image-lab-software?ID=KRE6P5E8Z

### Resource availability

#### Lead contact

Further information and requests for resources and reagents should be directed to and will be fulfilled by the lead contact, Jiao Liu (email: 2018683073@gzhmu.edu.cn).

#### Materials availability

This study did not generate new unique reagents.

#### Data and code availability

• RNA sequencing data have been deposited in the NCBI-SRA (NIH, USA) under accession no. PRJNA1040747 and are publicly available as of the date of publication.
• This paper does not report original code.
• Any additional information required to reanalyze the data reported in this paper is available from the lead contact upon request.

### Experimental model and study participant details

#### Cell lines

Human PDAC cell lines SW1990 (CRL-2172, male), PANC1 (CRL-1469, male), and MIAPaCa2 (CRL-1420, male) were procured from the American Type Culture Collection. These cells were cultured in Dulbecco’s modified Eagle’s medium (DMEM; Thermo Fisher Scientific, 11995073) supplemented with 10% fetal bovine serum (Thermo Fisher Scientific, A3840001) and 1% streptomycin/penicillin (Yeasen, 60162ES76) at standard conditions: 37°C temperature, 95% humidity, and 5% CO_2_.

All cell lines underwent mycoplasma testing and were authenticated via short tandem repeat profiling. When administering drugs or compounds, we prepared stock solutions for each using dimethyl sulfoxide (DMSO). The DMSO concentration in the working solutions for cell treatment remained below 0.01%. Throughout all cell culture assays, a DMSO concentration of 0.01% served as the control vehicle.

#### Mice

We conducted all animal care and experiments in accordance with the Association for Assessment and Accreditation of Laboratory Animal Care guidelines (http://www.aaalac.org) and with approval from our institutional animal care and use committee (University of Texas Southwestern Medical Center [102605] and Guangzhou Medical University [S2023-786]). All experimental and control animals were matched on sex and age. None were excluded from analysis at the time of harvest.

To develop murine subcutaneous tumors, we injected 5 × 10^6^ PANC1 cells into the flanks of NOD-SCID recipient mice (8–10 weeks old, female or male [1:1]).^69^^,^^70^^,^^71^ Treatment began when the tumors reached a size ranging from 150 to 200 mm^3^, typically around day 10 post-inoculation. The treatment regimens consisted of administering one of the following options: 1) A control vehicle; 2) CPC alone (2.5–10 mg/kg orally, once daily, 5 days per week); 3) CPC (10 mg/kg orally, once daily, 5 days per week) in combination with 4μ8C (20 mg/kg orally, once daily, 5 days per week); 4) CPC (10 mg/kg orally, once daily, 5 days per week) in combination with GS4997 (10 mg/kg orally, once daily, 5 days per week); and 5) CPC (10 mg/kg orally, once daily, 5 days per week) in combination with doramapimod (20 mg/kg orally, once daily, 5 days per week). These treatment regimens spanned a period of 3 weeks. We measured tumor volumes weekly using the formula: length × width^2^ × π/6. Additionally, we generated patient-derived organoids from PDAC patient liver metastases and subsequently expanded them as PDXs in NOD-SCID mice.^72^

To establish orthotopic tumors, female C57BL/6J mice (8–10 weeks old) underwent surgical implantation of either 5 × 10^5^ KPC cells into the tail of the pancreas.^73^^,^^74^^,^^75^ Ten days after the implantation, we randomly assigned the mice to different treatment groups and initiated a 3-week treatment regimen. During this period, the mice received one of the following treatments: 1) A control vehicle; 2) CPC alone (10 mg/kg orally, once daily, 5 days per week); 3) CPC (10 mg/kg orally, once daily, 5 days per week) in combination with 4μ8C (20 mg/kg orally, once daily, 5 days per week); 4) CPC (10 mg/kg orally, once daily, 5 days per week) in combination with GS4997 (10 mg/kg orally, once daily, 5 days per week); and 5) CPC (10 mg/kg orally, once daily, 5 days per week) in combination with doramapimod (20 mg/kg orally, once daily, 5 days per week). We monitored the survival of the animals on a weekly basis.

KPC mice were obtained and bred following the protocol established by Dr. David Tuveson at Cold Spring Harbor Laboratory.^76^ At 6 weeks of age, we randomly distributed both female and male mice in a 1:1 ratio into their respective groups. Subsequently, we assigned the treated mice to their respective groups using random allocation, and we initiated a 6-week treatment regimen. During this designated period, the mice received treatments that included either a vehicle or CPC (10 mg/kg orally, once daily, 5 days per week) in the absence or presence of 4μ8C (20 mg/kg orally, once daily, 5 days per week), GS4997 (10 mg/kg orally, once daily, 5 days per week), or doramapimod (20 mg/kg orally, once daily, 5 days per week). We monitored the survival of the animals on a weekly basis.

### Method details

#### RNA-seq

RNA was extracted from cells using the TRIzol reagent kit (Invitrogen, 15596026) following the manufacturer’s instructions. Each sample utilized 1 μg of RNA as input material for RNA sample preparations. Sequencing libraries were prepared using the NEBNext Ultra RNA Library Prep Kit for Illumina (NEB, E7530L), following the manufacturer’s recommendations, and index codes were added to associate sequences with individual samples. The library’s RNA concentration was quantified with the Qubit RNA Assay Kit on a Qubit 3.0 instrument and then diluted to 1 ng/mL. Insert size was assessed using the Agilent Bioanalyzer 2100 system (Agilent Technologies), and the accurately quantified insert size was confirmed using the StepOnePlus Real-Time PCR System (library valid concentration of >10 nM). Index-coded sample clustering was performed on a cBot cluster generation system using the TruSeq PE Cluster Kit v3-cBot-HS (Illumina), following the manufacturer’s instructions. After cluster generation, the libraries were sequenced on an Illumina Novaseq platform, generating 150 bp paired-end reads.^77^^,^^78^

#### Western blot

Cell lysates were prepared using a radioimmunoprecipitation assay buffer (Biosharp, BL509A), which was supplemented with a phosphatase inhibitor cocktail (Cell Signaling Technology, 5872).^79^^,^^80^^,^^81^ The cell lysis process was conducted on ice for 30 min, with intermittent shaking every 10 min, totaling three cycles. Following centrifugation at 15,000*g* for 15 min at 4°C, the resulting supernatants were collected and quantified using a bicinchoninic acid assay (BCA; Thermo Fisher Scientific, 23225).

A protein quantity of 30 μg was loaded onto 10% polyacrylamide gel electrophoresis gels (Epizyme, PG112) for the purpose of separation. Subsequently, proteins were transferred onto polyvinylidene difluoride membranes (Millipore, IPVH00010). After being blocked with 5% BSA, the membranes underwent overnight incubation at 4°C with primary antibodies (using the recommended dilution ratio per guidelines). This was followed by treatment with a horseradish peroxidase-labeled secondary antibody. The resulting signals were visualized using enhanced chemiluminescence (Thermo Fisher Scientific, 34095). Analysis of the blots was carried out using the ChemiDoc Touch Imaging System (Bio-Rad) in conjunction with Image Lab software (Bio-Rad).

#### The qPCR analysis

Total RNA isolation was carried out using the Cell Total RNA Isolation Kit (FOREGENE, RE-03111) per the manufacturer’s guidelines. Subsequently, first-strand cDNA was synthesized using 1μg of RNA with Hifair V one-step RT-gDNA digestion SuperMix (Yeasen, 11142ES60). For the cDNA synthesis, 20 μL reaction volumes were prepared by combining 4 μL of 5× Hifair one-step RT SuperMix, 2 μL of total RNA, and 1 μL of gDNA Remover Mix, which was diluted in 13 μL of RNase-, DNase-, and genomic DNA-free water.^71^^,^^82^

A quantitative real-time qPCR analysis was conducted utilizing Hieff qPCR SYBR Green Master Mix (Yeasen, 11201ES08) on a C1000 Touch Thermocycler CFX96 Real-Time System (Bio-Rad). The analysis was performed using Bio-Rad CFX Manager software 2.0. Normalization of the data was achieved using RNA 18S, and the fold change was calculated employing the 2^−ΔΔCt^ method.^83^^,^^84^ The primers used were as follows: *18S*: forward 5′-CTACCACATCCAAGGAAGCA-3′ and reverse 5′-TTTTTCGTCACTACCTCCCCG-3'; *HSPA5*: forward 5′-CTGTCCAGGCTGGTGTGCTCT-3′ and reverse 5′-CTTGGTAGGCACCACTGTGTTC-3'; *ATF6*: forward 5′-CAGACAGTACCAACGCTTATGCC-3′ and reverse 5′-GCAGAACTCCAGGTGCTTGAAG-3'; and *ERN1*: forward 5′-CCGAACGTGATCCGCTACTTCT-3′ and reverse 5′-CGCAAAGTCCTTCTGCTCCACA-3'.

#### RNAi and plasmid transfection

For siRNA transfection, cells were plated in 6-well plates at 60% confluence. In tube A, 150 μL of Opti-MEM and 7 μL of the siRNA were combined, while in tube B, 150 μL of Opti-MEM and 7 μL of siRNA-Mate transfection reagent (Genepharma, G04002) were mixed. After mixing tubes A and B, the solution was allowed to incubate for 20 min before being added to the 6-well plate. Cells were ready for experimentation 48 to 72 h post-transfection. The designed siRNAs targeting *ERN1* (#1 CCCAUCAACCUCUCUUCUGUA and #2 GAGAAGAUGAUUGCGAUGGAU) and a scramble control siRNA (UUCUCCGAACGUGUCACGUTT) were synthesized by Genepharma.

Human *EIF2AK3*-shRNA (CCTCAAGCCATCCAACATATT), *ATF6*-shRNA (CCCAGAAGTTATCAAGACTTT) and *ATG7*-shRNA (GCCTGCTGAGGAGCTCTCCAT) were sourced from IGE Biotechnology. GFP-MAP1LC3B1 lentiviral particles were purchased from Genechem. To generate high-titer lentiviral particles, 293FT cells (Thermo Fisher Scientific, R70007) were employed. Briefly, 293FT cells were pre-incubated with Opti-MEM reduced serum medium (Gibco, 31985070) for 1 h and then co-cultured with a mixture (shRNA [1600 ng], pSPAX2 [1200 ng], and pMD2G [400 ng]) for 8 h.^85^ Subsequently, the medium was replaced with DMEM, and the lentiviral particles were harvested 24–48 h later. The recipient cells were transduced with the lentiviral particles containing the specified shRNA for 24 h, followed by selection with puromycin (2 μg/mL; YEASEN, 60210ES72) for 7 days.

#### Cytotoxicity assay

For the evaluation of cytotoxicity, cells were seeded in 96-well plates at a density of 1 × 10^4^ cells per well and incubated overnight. Cytotoxicity was quantified using a CCK-8 Cell Proliferation and Cytotoxicity Assay Kit (Solarbio, CA1210) following the manufacturer’s instructions.^86^ In each well, the existing medium was replaced with 100 μL of fresh medium supplemented with 10 μL of CCK-8 solution. The cultures were then incubated in the cell incubator for 30–60 min at 37°C. The absorbance measured at 450 nm is proportionate to the viable cell count in the culture.

#### Colony formation assay

Cells were initially seeded in 12-well plates at a concentration of 800 cells per well and incubated for 24 h.^74^ Following 24 h of CPC treatment, the medium was replaced with fresh culture medium and maintained for 10 days. Subsequently, the cells were washed with a PBS solution, and the resulting colonies were visualized using 4% crystal violet staining (Solarbio, C8470). Quantitative analysis of the colonies was performed using ImageJ software, where the untreated knockdown group served as the reference set at 100% or 1. The cell cloning percentage was calculated as the ratio of the number of cell colonies in each well to the number in control wells.

#### Cell death assay

Cells were seeded at a density of 2.5 × 10^5^ cells per well in a medium within 6-well plates and allowed to incubate for 24 h. Following treatment with the specified reagents, cells were stained with Hoechst 33342 and PI (BestBio, BB-4131-1) for 30 min in a 5% CO_2_ incubator at 37°C.^87^^,^^88^ The cells exhibiting PI fluorescence were visualized using a fluorescence microscope (Carl Zeiss AG) at ×100 magnification. Quantitative analysis was conducted using ImageJ software, with the percentage of cell death calculated as the ratio of the PI signal to Hoechst 33342 signal for each well. In addition, a flow cytometric assay using Annexin V-FITC and PI staining was employed to measure apoptotic and necrotic cells.^89^ The term 'apoptosis rate' refers to the percentage of cells binding to Annexin V, indicating apoptosis.

#### Organelle-tracker staining

Cells were meticulously seeded onto specialized laser confocal petri dishes and allowed to adhere. Subsequently, the cells were incubated with ER-Tracker Red (Beyotime, C1041S), MitoTracker Green (Beyotime, C1048), and LysoTracker Green (Beyotime, C1047S) separately for 30 min at 37°C, ensuring protection from light to avoid photobleaching. Organelle-specific fluorescence patterns were captured utilizing a state-of-the-art confocal laser scanning microscope (Nikon A1+).

#### Thioflavin T staining

Cells were seeded at a density of 2.5 × 10^5^ cells per well in a medium within 6-well plates and allowed to incubate for 24 h. The medium was discarded then configured thioflacin T (1 μM) was added to the 6-well plate and incubated for 30 min at room temperature away from light. Cells showing green fluorescence (cells stained by thioflacin T) were observed using a Carl Zeiss AG fluorescence microscope (×100 magnification).

#### Transmission electron microscopy assay

Following the completion of drug treatments, cells were meticulously collected and then fixed with a solution of 2.5% glutaraldehyde, then underwent an overnight incubation at a temperature of 4°C. Following this fixation step, the samples underwent post-fixation for 60 min using a 1% osmium tetroxide solution.^90^ Subsequent to these fixation stages, the samples were meticulously embedded and sectioned. For enhanced visualization, the sections were subjected to a dual staining process involving uranyl acetate and lead citrate. Following this preparation, the sections were subjected to observation through a transmission electron microscope (FEI Company, Inc.), enabling detailed scrutiny of the ultrastructural features.

#### Immunofluorescence

For the immunofluorescence staining procedure, cells were initially rinsed with PBS and subsequently fixed using formaldehyde for a period of 30 min. Following fixation, cells were permeabilized utilizing Triton X-100, and nonspecific binding was blocked using 5% bovine serum for 1 h at room temperature. Cells were then subjected to an overnight incubation at 4°C with the primary antibody of interest. Subsequently, the cells were treated with an Alexa Fluor-conjugated secondary antibody for 1 h at room temperature. The resulting fluorescence signals were visualized using a fluorescence microscope (Carl Zeiss AG). For quantification purposes, the fluorescence intensity was semi-quantified utilizing ImageJ software.

### Quantification and statistical analysis

Data collection and analysis were performed using GraphPad Prism 8.02. Results were presented as mean ± standard deviation (SD), with *n* representing the number of independent repetitions. Unpaired Student’s *t* tests were employed to compare means between two groups. For comparisons among multiple groups, either one-way or two-way analysis of variance (ANOVA) with Tukey’s multiple comparisons test was used, as appropriate. Log rank tests were used to compare differences in mortality rates between groups. Statistical significance was established at *p* < 0.05.
