# Supplementary material for: Cetylpyridinium chloride triggers paraptosis to suppress pancreatic tumor growth via the ERN1-MAP3K5-p38 pathway
Source: iScience. 2024 Jul 26;27(8):110598. doi: 10.1016/j.isci.2024.110598 (PMC11357866; doi:10.1016/j.isci.2024.110598)
Supplement: Document S1. Figures S1–S3 [file mmc1.pdf]

iScience, Volume *27*

Supplemental information

Cetylpyridinium chloride triggers paraptosis to suppress pancreatic tumor growth via the ERN1-MAP3K5-p38 pathway

Hu Tang, Fangquan Chen, Wanli Gao, Xiutao Cai, Zhi Lin, Rui Kang, Daolin Tang, and Jiao Liu

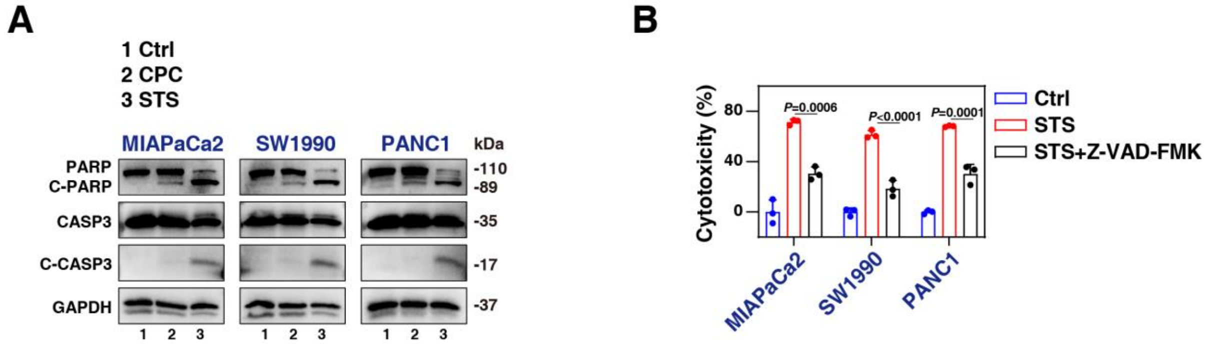

Figure S1. CPC induces non-apoptotic cell death in pancreatic cancer cells, Related to Figure 2.

(A) Western blot analysis of protein expression in MIAPaCa2, SW1990, and PANC1 cells after treatment with CPC (7.5 µM) or staurosporine (STS; 1 µM) for 24 h.

(B) Cytotoxicity of MIAPaCa2, SW1990, and PANC1 cells after treatment with STS (1 µM) in the absence or presence of Z-VAD-FMK (20 µM) for 24 h.

The data are presented as the mean ± SD from at least three independent experiments (B). *P* values were calculated by one-way ANOVA (B).

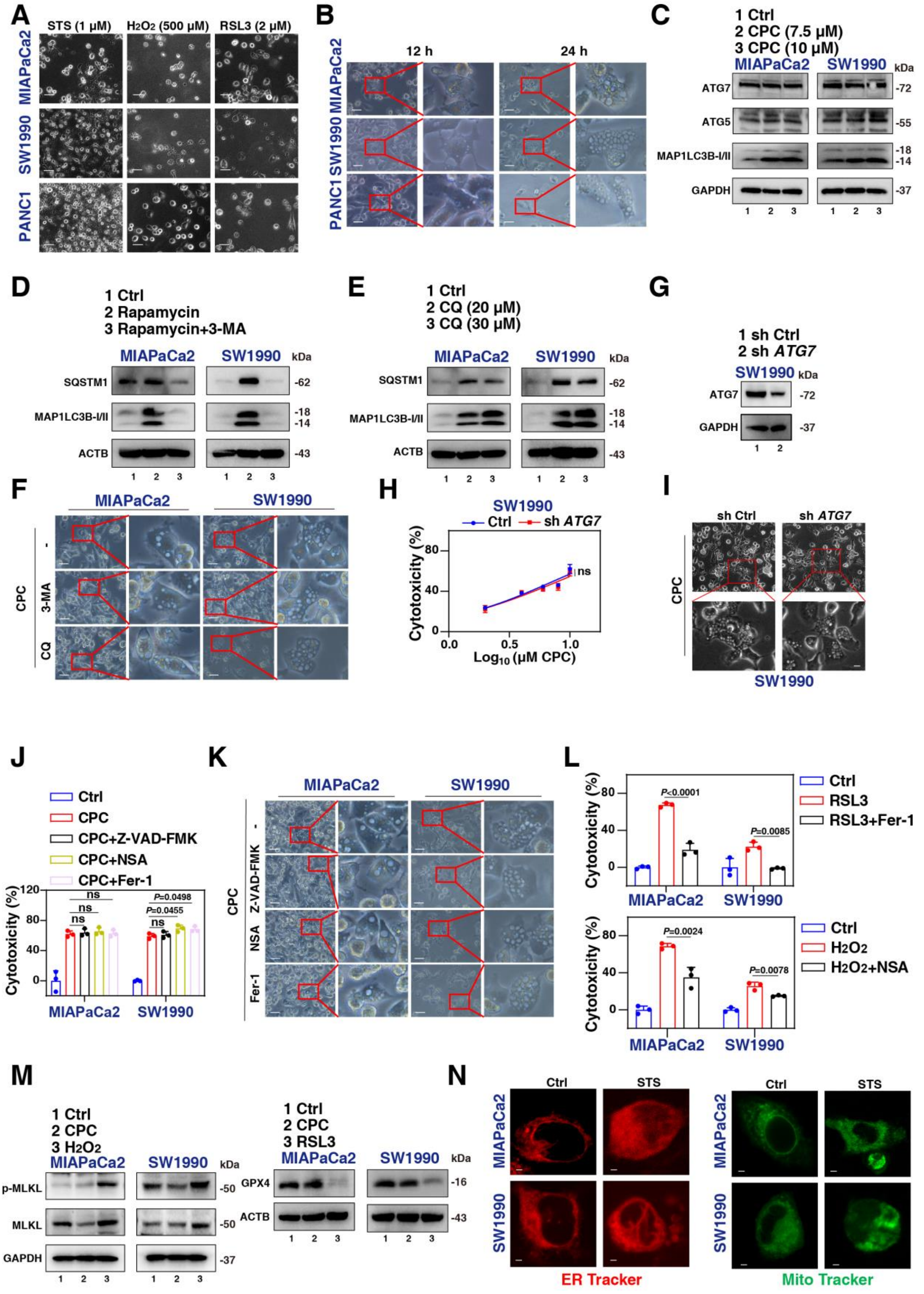

Figure S2. The cell vacuoles induced by CPC originated from the ER and mitochondria,

Related to Figure 3.

(A) Phase-contrast microscope analysis of cell morphology in MIAPaCa2, SW1990, and PANC1 cells after treatment with staurosporine (STS; 1 µM), H_2O_2 (500 µM) or RSL3 (2 µM) for 24 h. Scale bar: 50 µm.

(B) Light microscopy analysis of cell morphology in MIAPaCa2, SW1990, and PANC1 cells after treatment with CPC (7.5 µM) for 12 and 24 h. Scale bar: 50 µm.

(C) Western blot analysis of protein expression in MIAPaCa2 and SW1990 cells after treatment with CPC (7.5 and 10 µM) for 24 h.

(D) Western blot analysis of protein expression in MIAPaCa2 and SW1990 cells after treatment with rapamycin (5 µM) in the absence or presence of 3-methyladenine (3-MA; 3 mM) for 24 h.

(E) Western blot analysis of protein expression in MIAPaCa2 and SW1990 cells after treatment with chloroquine (CQ; 20 and 30 µM) for 24 h.

(F) Light microscopy analysis of cell morphology in MIAPaCa2 and SW1990 cells after treatment with CPC (7.5 µM) in the absence or presence of 3-methyladenine (3-MA; 3 mM) or chloroquine (CQ; 20 µM) for 24 h. Scale bar: 50 µm.

(G) Western blot analysis of protein expression in control and ATG7-knockdown SW1990 cells.

(H) The cytotoxicity of indicated SW1990 cells after treatment with CPC for 24 h.

(I) Phase-contrast microscope analysis of cell morphology in indicated SW1990 cells after treatment with CPC (7.5 µM) for 24 h. Scale bar: 50 µm.

(J) Cytotoxicity of MIAPaCa2 and SW1990 cells after treatment with CPC (7.5 µM) in the absence or presence of Z-VAD-FMK (20 µM), NSA (2 µM), or Fer-1 (1 µM) for 24 h.

(K) Light microscopy analysis of cell morphology in MIAPaCa2 and SW1990 cells after treatment with CPC (7.5 µM) in the absence or presence of Z-VAD-FMK (20 µM), necrosulfonamide (NSA; 2 µM) or ferrostatin-1 (Fer-1; 1 µM) for 24 h. Scale bar: 50 µm.

(L) Cytotoxicity of MIAPaCa2 and SW1990 cells after treatment with ferroptosis inducer RSL3 (2 µM) or necroptosis inducer H_2O_2 (500 µM) in the presence or absence of the corresponding inhibitors Fer-1 (1 µM) or NSA (2 µM) for 24 h.

(M) Western blot analysis of protein expression in MIAPaCa2 and SW1990 cells after treatment with CPC (7.5 µM), RSL3 (2 µM) or H_2O_2 (500 µM) for 24 h.

(N) MIAPaCa2 and SW1990 cells were treated with STS (1 µM) for 24 h. ER and mitochondrial structures were stained with ER-Tracker Red and Mito-Tracker Green, respectively. Scale bar: 10 µm.

Data are presented as mean ± SD. The proportion of cells displaying vacuoles was determined by visually examining a minimum of 100 cells, and the data are presented as the mean ± SD from at least three independent experiments (A, B, F,I, K). *P* values were calculated by one-way ANOVA (J and L) or two-way ANOVA (H).

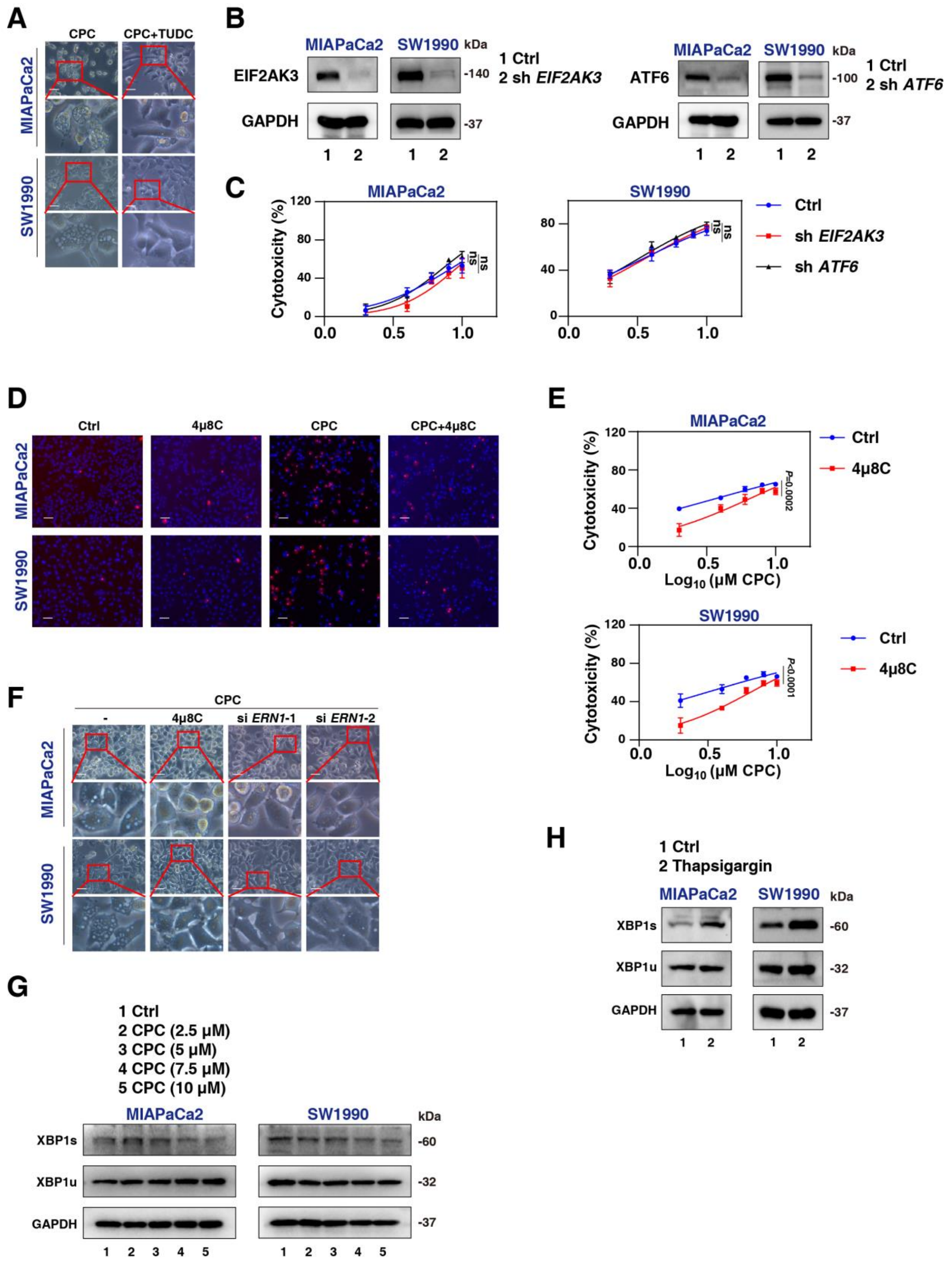

Figure S3. ERN1 promotes paraptosis and activates MAP3K5, Related to Figure 5 and 6.

(A) Light microscopy analysis of cell morphology in MIAPaCa2 and SW1990 cells after treatment with CPC (7.5 µM) in the absence or presence of tauroursodeoxycholate (TUDC; 1 mM) for 24 h. Scale bar: 50 µm.

(B) Western blot analysis of protein expression in control and indicated *EIF2AK3*- or *ATF6*-knockdown PDAC cells.

(C) The Cytotoxicity of indicated MIAPaCa2 and SW1990 cells after treatment with CPC for 24 h.

(D) Representative Hoechst 33342 and PI staining images of indicated MIAPaCa2 and SW1990 cells after treatment with CPC (7.5 µM) in the absence or presence of 4µ8C (100 µM) for 24 h. Scale bar: 200 µm.

(E) Cytotoxicity of MIAPaCa2 and SW1990 cells after treatment with CPC in the absence or presence of 4µ8C (100 µM) for 24 h.

(F) Representative light microscopy images of indicated MIAPaCa2 and SW1990 cells after treatment with CPC (7.5 µM) in the absence or presence of 4µ8C (100 µM) for 24 h. Scale bar: 20 µm.

(G) Western blot analysis of protein expression in MIAPaCa2 and SW1990 cells after treatment with CPC for 24 h.

(H) Western blot analysis of protein expression in MIAPaCa2 and SW1990 cells after treatment with ER stress inducer thapsigargin (1 µM) for 24 h.

The proportion of cells displaying vacuoles was determined by visually examining a minimum of 100 cells, and the data are presented as the mean ± SD from at least three independent experiments (A, F). *P* values were calculated by two-way ANOVA (C, E).

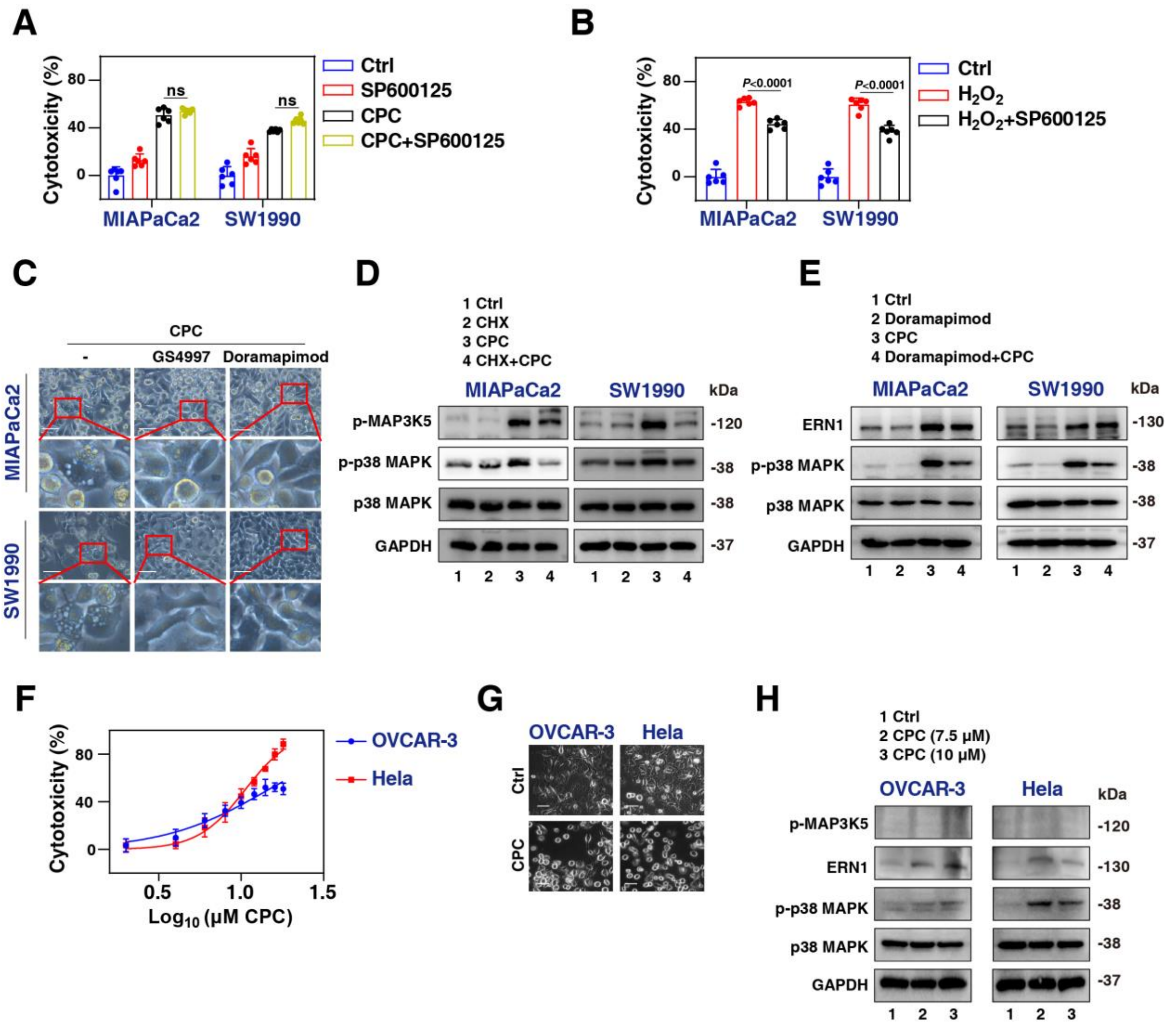

Figure S4. Evidence that p38 is critical for CPC-induced paraptosis, , Related to Figure 7.

(A) Cytotoxicity of MIAPaCa2 and SW1990 cells after treatment with CPC (7.5 µM) in the absence or presence of SP600125 (20 µM) for 24 h.

(B) Cytotoxicity of MIAPaCa2 and SW1990 cells after treatment with H_2O_2 (500 µM) in the absence or presence of SP600125 (20 µM) for 24 h.

(C) Representative light microscopy images of MIAPaCa2 and SW1990 cells after treatment with CPC (7.5 µM) in the absence or presence of GS4997 (10 µM) or doramapimod (5 µM) for 24 h. Scale bar: 20 µm.

(D, E) Western blot of protein expression in MIAPaCa2 and SW1990 cells after treatment with CPC (7.5 µM) in the absence or presence of cycloheximide (CHX; 5 µM) or

doramapimod (5 μM) for 24 h.

(F) Cytotoxicity of OVCAR-3 and Hela cells following treatment with CPC for 24 h.

(G) Phase-contrast microscope analysis of cell morphology in OVCAR-3 and Hela cells after treatment with CPC (7.5 and 10 μM) for 24 h. Scale bar: 50 μm.

(H) Western blot analysis of protein expression in OVCAR-3 and Hela cells after treatment with CPC for 24 h.

The proportion of cells displaying vacuoles was determined by visually examining a minimum of 100 cells, and the data are presented as the mean ± SD from at least three independent experiments (C, G). *P* values were calculated by one-way ANOVA (A, B).
